# Emotional processes, collective behavior, and social movements: A meta-analytic review of collective effervescence outcomes during collective gatherings and demonstrations

**DOI:** 10.3389/fpsyg.2022.974683

**Published:** 2022-08-31

**Authors:** José J. Pizarro, Larraitz N. Zumeta, Pierre Bouchat, Anna Włodarczyk, Bernard Rimé, Nekane Basabe, Alberto Amutio, Darío Páez

**Affiliations:** ^1^Faculty of Psychology, University of the Basque Country, San Sebastian, Spain; ^2^Escuela de Psicología, Universidad Católica del Norte, Antofagasta, Chile; ^3^Université de Lorraine, Équipe PerSEUS (EA 7312), Metz, France; ^4^Department of Psychology, Université catholique de Louvain, Ottignies-Louvain-la-Neuve, Belgium; ^5^Facultad de Educación y Ciencias Sociales, Universidad Andrés Bello, Santiago, Chile

**Keywords:** collective effervescence, Durkheim, emotions, social integration, social values and beliefs, empowerment, collective rituals and gatherings

## Abstract

In this article, we review the conceptions of Collective Effervescence (CE) –a state of intense shared emotional activation and sense of unison that emerges during instances of collective behavior, like demonstrations, rituals, ceremonies, celebrations, and others– and empirical approaches oriented at measuring it. The first section starts examining Émile Durkheim's classical conception on CE, and then, the integrative one proposed by the sociologist Randall Collins, leading to a multi-faceted experience of synchronization. Then, we analyze the construct as a process emerging in collective encounters when individuals contact with social ideal and values, referring to the classical work of Serge Moscovici as well as those more recent empirical approaches. Third, we consider CE as a set of intense positive emotions linked to processes of group identification, as proposed by authors of the Social Identity Theory tradition. Finally, we describe CE from the perspective of self-transcendence (e.g., emotions, experiences), and propose a unified description of this construct. The second section shows the results of a meta-analytical integration (*k* = 50, *N* = 182,738) aimed at analyzing CE's proximal effects or construct validity (i.e., Individual Emotions and Communal Sharing) as well as its association with more distal variables, such as Collective Emotions, Social Integration, Social Values and Beliefs and Empowerment. Results indicate that CE strongly associates with Individual Emotions –in particular, Self-Transcendent Emotions– and Communal Sharing constructs (e.g., Group Identity, Fusion of Identity), providing construct validity. Among the distal effects of CE, it is associated with Collective Positive Emotions, long-term Social Integration (e.g., Ingroup Commitment), Social Values and Beliefs and Empowerment-related variables (e.g., Wellbeing, Collective Efficacy, Collective Self-Esteem). Among the moderation analyses carried out (e.g., study design, CE scale, type of collective gathering), the effects of CE in demonstrations are noticeable, where this variable is a factor that favors other variables that make collective action possible, such as Group Identity (*r*_*pooled*_ = 0.52), Collective Efficacy (*r*_*pooled*_ = 0.37), Negative and Self-Transcendent Emotions (*r*_*pooled*_ = 0.14 and 0.58), and Morality-related beliefs (*r*_*pooled*_ = 0.43).

## Introduction

### Different approaches to collective effervescence

At the dawn of the twentieth century, a series of researchers and theorists tried to understand and explain the human experience in social rituals and gatherings (e.g., Freud, 1922; Le Bon, 2002). Since then, their work inspired significant lines of research including—among others—the creation of common identities (e.g., Tajfel and Turner, 1979) and even the birth of modern societies (e.g., Whitehouse et al., 2019; Henrich, 2020). Among this body of research, the work of Émile Durkheim has sketched the basis of a functional perspective of rituals and gatherings and, particularly, of a mechanism that produces psychosocial effects at different levels of analysis.

In his famous book, *Elementary forms of Religious Life* (1912/1915)—based on the ethnographies conducted by Spencer and Gillen (1898) with native tribes of Australia—Durkheim proposed an emotion-based mechanism capable of facilitating a series of social effects. As he conceived it (for a well-detailed analysis of his work, see Maryanski, 2018), this mechanism was key to understanding the evolution of social groups and the development of societies. Now, more than 100 years after this influential book, we present a review of theoretical models and empirical studies that have employed Durkheim's theoretical advances. In the following sections, we describe what they are, how they differ across theoretical perspectives, and present a meta-analysis of empirical studies that analyze the role of Durkheim's proposed mechanism and its effects on various psychosocial variables.

#### Durkheim's view of collective effervescence as intense shared emotions

For Durkheim (1912/1915), Collective Effervescence (hereafter, CE) was a process of synchronization and intensification of emotions among individuals that occurs during participation in collective rituals, and he considered it as a central component of collective behavior by which society empowers individuals to cope with the vicissitudes of life. In his view, if left alone, individuals would be unable to face existence and its intellectual challenges. CE thus starts with the effects of the mere gathering which brings individuals closer together, multiplies contacts between them, and makes them more intimate.

(…) their first effect is to bring individuals together, to multiply the relations between them, and to make them more intimate with one another. By this very fact, the contents of their consciousnesses are changed. (p. 348)

Under these conditions, the utilitarian and individual preoccupations that dominate in profane life are eclipsed and the parcel of social being that each person carries within is revived and emerges to the forefront of consciousness. Thoughts focus on common beliefs, common traditions, and collective ideals. Homogeneous manifestations then develop in the assembly. By uttering the same cries, the same words, and the same gestures, individuals nourish the group feeling. Strong emotional experiences arise because every feeling expressed resounds in other consciences. Each of them echoes the others and vice versa, resulting in a reciprocal amplification. Progressively, participants enter into communion and CE takes place. Durkheim argued that once individuals are assembled, a “sort of electricity is formed by their collecting which quickly transports them to an extraordinary degree of exaltation” (p. 212). He insisted that the CE is independent of the type of emotion involved. Whatever the emotion, the key point is that it has to be shared. The social sharing of emotions in itself represents the decisive condition for a state of effervescence to arise. This point is made particularly clear in the author's descriptions of mourning rituals which are framed around negative emotions (see also Turner and Stets, 2006). Durkheim described a social dynamic developing when individuals come together. First, participants' co-presence and interaction generate a cognitive change in which the individual consciousness gives way to group consciousness. Next, gestures, actions, and movements become homogeneous feeding up collective feelings. Third, the reciprocal amplification of expressed feelings yields emotional communion and collective effervescence. Participation in a collective gathering enhances participants' sense of social belonging.

The reciprocal stimulation of their emotions and the homogeneity of their gestures and movements lead them to feel in unison. Durkheim proposes four main outcomes of CE: first, CE is intrinsically related to the intensification and convergence of emotions and the creation of an emotional atmosphere or collective mood and emotions. Second, participation in a collective gathering enhances participants' sense of social belonging and by this token social cohesion. The reciprocal stimulation of their emotions and the homogeneity of their gestures and movements lead them to feel in unison. Third, by acting together, participants thus recreate the group consciousness. It brings common beliefs and collective representations to the foreground of thoughts. Fourth, the gathering of individuals entails exceptionally energizing effects, empowers them, and reinforces vital energy. Collective gatherings revitalize a collective part of consciousness that is latent in ordinary life. This part is made of shared representations. Their reactivation recreates the unity of participants' consciousness.

As is seen in the following sections, Durkheim's work can be taken as the starting point and inspiration for many theorists and researchers who have tested and expanded his conceptions of CE. While his views could be considered generic and even poetic at times, further developments have greatly extended the theoretical conception of CE and its social effects.

### Approaches to collective effervescence

#### Collins' interaction ritual theory

Collins' (2004) developed an interaction ritual theory inspired by Goffman's (1959) study of interaction rituals and then, extended his perspective to larger groups as explored by Durkheim's (1912/1915) theory of collective rituals. For Collins (2004), the development of social life rests on two preconditions. First, human bodies need to be assembled in the same place and affect one another and next, their mere co-presence should be converted into focused interactions. Once a mutual focus of attention develops, a “shared reality” becomes effective among coparticipants. Indeed, once the bodies are together, there may take place a process of intensification of shared experience which Durkheim called CE, and the formation of a collective conscience or consciousness. We might refer to it as a condition of heightened intersubjectivity, which, according to Collins, rests on two mutually reinforcing elements: shared actions and shared emotion.

Collins described CE as a transitory state with sustained effects. Four outcomes result from the experience of heightened mutual awareness and emotional arousal. First, group emblems, the markers of group identity, are shaped. Durkheim (1912/1915) argued that devoid of symbols, sentiments have only a precarious existence. Second, ideals and values are consecrated. Rituals thus charge symbolic objects with new significance or recharge them with renewed sentiments of respect. Third, as individual participants are also recharged in this process, individual energy is produced named by Collins “emotional energy”. The final effect of rituals' outcomes is morality. When people act under the energy derived from the heightened experience of intersubjectivity and emotional strength, they feel moral: “It is a morally suffused energy; it makes the individual feel not only good but exalted, with the sense of doing what is most important and most valuable” (p. 39).^1^

#### Contact with values and self-transcendent beliefs

Serge Moscovici (1988/1993) shared Durkheim's view that individuals in isolation lack vital energy. Recent empirical evidence largely supports this view by documenting relationships linking social isolation, poor health, and low wellbeing (e.g., Larson, 1990; Holt-Lunstad et al., 2015; Liu et al., 2018). These new data give particular significance to the view that people replenish themselves when members of a society are in communion in feeling, thought, and action. Moscovici (1988/1993) argued that when in groups and collective situations, individuals converge and polarize their beliefs, emotions and behaviors. Individual borders are then blurred and participants let their emotions flow but they do not lose their capacity to reason. Collective gatherings prompt a shift from an initial state in which individuals are turned inward to a subsequent state in which they communicate and merge. They harmonize their feelings and representations with those of their coparticipants.

Durkheim (1912/1915) contended that *Homo sapiens* is a *Homo duplex*, or a creature living on two levels, with an individual and a part of the broader society. Moscovici (1988/1993) viewed this dual nature of human beings as essential. Taking part in a ceremony makes people realize that each of them represents both an individual and a collective being. They live in two worlds, the profane—or world of daily life—and the sacred—or world of ideal values. CE is what brings these two worlds together. Other psychologists also conceive CE as a positive emotional experience generated during collective gatherings, resulting from a feeling of sacredness arising from participating in them (Gabriel et al., 2020) and of a motivational disposition to enjoy participating (Gabriel et al., 2017). Thus, Gabriel et al. (2020) operationalized CE as a sense of connectedness—assessed through items such as “I felt connected to others who were present at the event”, “the event made me feel closer to the people who were there”)—associated with a feeling of sacredness (e.g., “I felt as if there was something sacred about the event”).

#### Perceived emotional synchrony

In line with Durkheim's views on the contribution of participants' synchronicity to the emergence of CE, Páez et al. (2015) stressed that in a mass event, people experience a multifaceted synchronization with co-participants. They share time and place, concerns (e.g., shared intentions, goals, purposes), attentional focus (e.g., podium, stage, altar, speaker, leader, priest), actions (e.g., gestures, movements, marching), expression (e.g., singing, yelling, repeating sentences, playing music, dancing), as well as emotional responses to the shared situation. The combined effects of these various elements of synchronicity stimulate participants' experience and enactment of similar emotional states, thus fueling an experience of fusion or unison, which Durkheim often referred to as emotional communion. Páez et al. (2015) considered that assessing participants' subjective experience of these combined components would provide an empirical proxy for the Durkheimian notion of CE. The proposed variable was labeled Perceived Emotional Synchrony (hereafter, PES) and defined as an emotional experience felt by participants during group gatherings and involving a sense of togetherness. It addresses not only the experience of emotions felt together but also the collective synchronization of all the various facets of the emotional experience. Example items used were “We felt more sensitive to emotions and feelings that others feel,” “We felt a strong-shared emotion,” or “We performed as one, like a single person” (see Wlodarczyk et al., 2020). Different studies examined the effects of participation in different types of collective gatherings. Participation and specifically PES strengthened social integration, self-esteem, positive affect, and socially shared beliefs (Páez et al., 2015; Pelletier, 2018; Bouchat et al., 2020; Wlodarczyk et al., 2020, 2021; Zumeta et al., 2020; Kettner et al., 2021).

#### Positive emotions stemming from shared social identity

CE has also been conceptualized and empirically measured by several social psychologists working within the framework of Social Identity Theory (Tajfel and Turner, 1979) and its development in Self-Categorization Theory (Turner et al., 1987)—hereafter, the Social Identity Perspective (SIP, see Hornsey, 2008). They suggest (e.g., Hopkins et al., 2016) that CE corresponds to a feeling of strong positive emotions that potentially follows once a sense of shared identity develops amongst participants at meetings, demonstrations, or collective rituals. First, in a cognitive change, individuals stop thinking of themselves in terms of their personal identities and start viewing themselves as members of a common category. Values and beliefs associated with the social identity salient at the time become the keys to appraising their current situation. Secondly, a relational change arises as participants develop a sense of connection and intimacy with co-participants (Neville and Reicher, 2011). Third, an affective change develops, as emotions are no longer based upon personal considerations but upon social identity-related ones. These include appraisals of stimuli and the experience of relational intimacy described above. Additionally, the sense of empowerment felt in crowds and the consequent ability to achieve group goals may be a basis for the strong positive emotions often found in crowds (Drury and Reicher, 2005; Hopkins et al., 2016; Stott et al., 2018). These strong positive emotions are viewed by these authors as being similar to the concept of CE (e.g., Hopkins et al., 2016). This line of research empirically assessed CE through participants' ratings of how positive their collective experience was (e.g., “In the period of pilgrimage, to what extent have you felt fulfilled, happy, and so on?”), or in terms of the experience of positive emotions (e.g., “I felt excited and I felt cheerful at …”) (Novelli et al., 2013). In rituals, demonstrations, or meetings, different studies recorded positive and significant associations between identity-related processes and intense positive emotions—or, in their view, CE—(Novelli et al., 2013; Hopkins et al., 2016; Alnabulsi et al., 2020).

#### Self-transcendent emotions

In CE, several elements contribute to bringing participants beyond the world of their ordinary experience. For instance, people perceive that they share emotions with others, which reinforces their collective identity and empathy with group members, and attention is thus directed outwards so that self-absorption drops. In addition, stimuli arising out of other-focused appraisals or other-directed attention (e.g., others' suffering, virtues, love, or closeness) as theorized above (i.e., Collins, 2004; Páez et al., 2015; Hopkins et al., 2016) are common elicitors of a subset of positive emotions, referred to as “moral emotions,” “other-praising emotions,” or “self-transcendent emotions.” They include elevation, compassion, admiration, gratitude, love, and awe (Haidt, 2003a,b; Algoe and Haidt, 2009; Haidt and Morris, 2009; Van Cappellen and Rimé, 2014).

These emotions decrease the salience of the individual self and promote union with other people and social groups (Haidt, 2003b; Van Cappellen and Rimé, 2014; Stellar et al., 2017). They mobilize people to connect with those around them or with society and thus foster episodes of self-transcendence. Such episodes involve not only a decrease in self-absorption, but also the blurring of the boundaries separating the individual from the environment, the interpenetration of the individual self and the group, and a broader connection with the world (Van Cappellen and Rimé, 2014; Yaden et al., 2017; Hanley and Garland, 2019). Therefore, CE could be viewed as a self-transcendent emotion (Haidt et al., 2008), or as a manifestation of a mode of sociality that represents union (e.g., Fiske, 1992).

Indeed, the latter is what Fiske et al. (2017) proposed, suggesting that Durkheim's CE is a manifestation of the mode of relationship Communal Sharing (i.e., horizontal and egalitarian social relationships based on strong bonds; see Fiske, 1992). In addition, and when communal sharing relationships suddenly intensify, they produce an analog of a strong emotional state, which they term *kama muta* (in Sanskrit meaning “moved by love”). In their view, cultural practices such as collective gatherings and rituals can evoke in witnesses and participants the sudden intensification and salience of the Communal Sharing mode. In turn, this triggers kama muta, characterized by feeling moved or touched, positive affect, bodily responses such as tears, chills, or warmth, and action tendencies such as approach behavior, affiliation, prosocial behavior, and social bonding (Zickfeld et al., 2019a,b). Supporting this, there are different studies showing strong positive correlations between with self-transcendent emotions (Zumeta et al., 2020; Wlodarczyk et al., 2021).

### Toward a working definition of collective effervescence

After this review, we propose CE as a state of intense and joint emotional activation, which can potentially emerge in instances of collective behavior and can generate a series of effects at the individual (e.g., wellbeing) and collective (e.g., collective identity and values) levels. It is a process that implies attentional (i.e., shared focus of attention) and behavioral (i.e., coordination of movements and gestures) convergence and, above all, emotional synchronization (i.e., a convergence of different emotional components). When these criteria are met, CE usually implies a beyond-normal emotional intensification or emotional feedback. In other words, after the convergence of attention, as well as that behavioral and emotional, participants of a collective gathering will feel emotions of greater-than-normal intensity and an enhanced sense of unison with others.

#### Outcomes of collective effervescence

Here, we briefly summarize the major effects of CE that recur among authors (e.g., Durkheim, 1912/1915; Collins, 2004; Páez et al., 2015; Hopkins et al., 2016; Zumeta et al., 2020). In addition, the effects listed here will then be adopted as criterion variables in our meta-analytic review of studies, which assessed the experience of collective effervescence in collective events. While the dimensions of the variables included here are six (see below), we organize them into proximal and distal outcomes (see Section Method):

Proximal outcomes of CE include:

(1) Affective reactions or the emotions felt by participants as individuals during collective gatherings. These emotions—which are proximal from a time perspective—include (a) general activation or emotional arousal; (b) negative emotions in negatively valenced events; (c) positive emotions, which are often reported when individuals come together even when their meeting involves negative emotions (e.g., funerals); (d) self-transcendent positive emotions which include elevation, compassion, admiration, gratitude, kama muta or moved by love, and social awe;
(2) Communal sharing. These proximal effects refer to the individual relationship with her co-participants of the collective gatherings and includes (a) social identification or self-categorization as a member of the group or collective; (b) feeling the individual self as less important than the collective self, or feeling that the individual self and the collective overlap, merge, or are fused into one; and (c) an increased sensation of social support with the group.

Outcomes that are more distal may include:

(3) Collective emotions or emotions perceived as dominant in the group during a given period, and organized broadly into negative or positive climate.
(4) Social integration. This group of outcomes consists on those variables that reflect a psychological connection with those represented in the gathering but not necessarily present. It includes: (a) an enhanced feeling of commitment toward the group, and (b) an identification with those that are not in the gathering (e.g., with the whole movement of women and not only with co-participants in the 8M demonstrations; see below).
(5) Social values and beliefs. According to what is being enacted, represented, celebrated, etc., in the collective gathering, CE can facilitate greater agreement with (a) social values (e.g., self-transcendent values such as universalism) and (b) self-transcendent beliefs, spirituality- and religion-related beliefs, as well as those related to purpose or meaning of life.
(6) Empowerment among those who participate. Finally, CE can boost the subjective perception of (a) vitality and wellbeing among those who take part in the collective event, as well as (b) self-esteem and efficacy. The latter, could be related to co-participants (e.g., women who attend to 8M demonstrations) or to an extended group (e.g., all women in the world).

#### Demonstrations, collective effervescence and social movements

Specifically, in order to analyze the role of CE in the fueling of social movements, we will examine its association with factors that are both conducive and explanatory of the participation in collective action or social movements. Demonstrations as a protest ritual are not only aimed to change the social milieu, but are also symbolic performance with an expressive purpose. Demonstrations “provide participants with the sense of being engaged in a common cause with a large number of like-minded people who share similar feelings about an issue, mass gatherings also work as opportunities to cement a given social group” (Casquete in Filleule and Tartakowski, 2013). “In the midst of an assembly” Durkheim (1912/1915) writes, “we become capable of feelings and conduct of which we are incapable when left to our individual resources.” “[…] For this reason all parties—be they political, economic, or denominational—see to it that periodic conventions are held, at which their followers can renew their common faith by making a public demonstration of it together.” (p. 212).

Demonstrations, therefore, are opportunities for constructing or reinforcing group solidarity and identity as well as ritual occasions with socializing effects. As Filleule and Tartakowski (2013) posit, participation in demonstrations are opportunities for constructing or reinforcing solidarity and collective identity as well as ritual occasions with socializing effects, and should reinforce factors conducive to collective action. Meta-analytical reviews support that factors conducive to collective action are social or collective identification (i.e., identification with an extended group; van Zomeren et al., 2008; Agostini and van Zomeren, 2021; Akfirat et al., 2021), collective efficacy (van Zomeren et al., 2008; Agostini and van Zomeren, 2021), negative emotions related to affective fraternal or collective deprivation (van Zomeren et al., 2008; Smith et al., 2012), agreement with self-trascendence values, that could reinforce moral conviction supportive of collective action (Sabucedo et al., 2018; Agostini and van Zomeren, 2021) as well as moral, positive and self-transcendent emotions like hope, that gave motivational support to the previous factors (Agostini and van Zomeren, 2021). Therefore, a clear hypothesis is that the participation in social and political collective protests should increase the level of these factors favorable to collective action.

#### Differentiation with related constructs: Collective emotions and co-experienced emotions

As it can result evident, the presented definition of CE can overlap or be similar to other affective phenomena. Therefore, the following lines will attempt to differentiate CE from similar constructs, such as collective emotions, or co-experienced emotions.

One of the effects that can result from CE over time is the creation of a collective affective state or collective emotion (Collins and Hanneman, 1998; von Scheve, 2011; see also Thonhauser, 2022). Collective emotions are the convergence of an affective response felt by two or more people (i.e., a collective) toward a specific event or object (von Scheve, 2011). Thus, participation and interaction in collective events have a large implication (e.g., through CE), but collective emotions correspond to a larger phenomenon than CE. Collective emotions are not just a convergence of affect or shared emotions. Rather, they are the result of a series of characteristics and involve—to varying degrees—a culture of emotional norms, being shared by a large proportion of people, being “distributed” in collective gatherings, and more (see von Scheve, 2011; Basabe and Paez, 2017). Because they originate from cultural values and norms of a collective (e.g., a common evaluative perspective, based on a history of previous interactions; see Thonhauser, 2022), these can have a normative and prescriptive value, and can impose on individuals what is socially desirable (von Scheve, 2011; Menges and Kilduff, 2015; Basabe and Paez, 2017). As an example, one group of people celebrating the origins of a national celebration could, through joint emotional activation and synchronization during the event (i.e., CE), experience intense proud and joy in front of the national symbols. In turn, and over time, these experiences can boost a common and shared interpretation of what should be enacted and felt during a national celebration and thus, national institutions (e.g., government, schools) can further “teach” what is supposed to be felt during a national celebrations (i.e., collective emotion). As it can be seen, collective emotions imply top-down dynamics, are larger in scope, and can be the cause or result of CE, which in turn, in comparison, corresponds to a smaller-in-scope process.

On the other hand, emerging research argues that co-experienced positive affect is centered on “love-the-emotion” (Fredrickson, 2016). This proposal aims at defining a social emotion (i.e., different from love-the-sentiment or love-the-attitude) and presents the criteria that produce it: rapport or mutual awareness of the shared positive emotion and bio-behavioral synchronization. In other words, Fredrickson (2016) proposes that this emotion is felt in any instance of shared positive emotion when the above criteria are met (Fredrickson, 2016; Brown and Fredrickson, 2021). In addition to this view, CE can be also thought of as a manifestation of the communal sharing mode of social relationships (Fiske, 1992). According to this theory, however, when this mode of sociality suddenly intensifies (e.g., a reunion of a couple of lovers after being separated) produces a social emotion called kama muta (see Fiske et al., 2017).

Our conceptualization of CE greatly overlaps with these two. However, it is important to clarify that CE is not a particular emotion (i.e., neither love-the-emotion nor kama muta), but rather an emotional phenomenon that implies jointly experienced affect. In addition, it is not subjected to positive affect solely; rather, it can also be the result from negatively valenced emotions, such as the common pain, sadness and grief present at a funerary ritual.

### Overview of the present meta-analysis

So far, we have reviewed theories and research related to CE. We will now consider available empirical studies and datasets addressing collective events and report a meta-analytic examination of the relationship between their various measures and operationalizations of this construct, as well as its relationship with several criterion variables. For the latter, we considered variables that came up repeatedly throughout the review of the theoretical and empirical literature. The meta-analytic review will allow an accurate assessment of the association of each of these variables with measures of CE.

Accordingly, we organize the associations of CE with a series of dependent variables from empirical studies, similarly as presented above (i.e., affective responses, communal sharing, values and beliefs, and empowerment) and in two levels of analyses: proximal and distal outcomes (see Section Procedure). In further detail, we examine the effects of participation in demonstrations to examine whether they reinforce explanatory principles of social movements, such as collective or social identity, social efficacy, negative emotions linked to relative deprivation and injustice, as well as positive moral emotions.

## Method

### Procedure

Following the American Psychological Association (APA) Meta-Analysis Reporting Standards (MARS) and more biological related PRISMA guidelines (Moher et al., 2015), we conducted several searches between December 2019 and March 2022 in PsycINFO, WoS, SCOPUS (all in English) and Google Scholar (in English, French, Italian, Portuguese, and Spanish). The key search terms were: “Collective Effervescence,” “Effervescence and Durkheim,” “Emotional Effervescence,” “Emotional Synchrony and Collective,” and “Collective Emotions” (and equivalent terms in languages other than English). A final search was conducted in Google Scholar using the same terms, always including the word “Durkheim”. Full datasets, R-code syntax, and Supplementary material (i.e., including further moderation analyses) can be freely accessed at our project's online repository of Open Science Framework: https://osf.io/wb8c5/?view_only=~da9c179483f64d229a29ff5397bb9930.

#### Inclusion criteria

**Figure 1:**
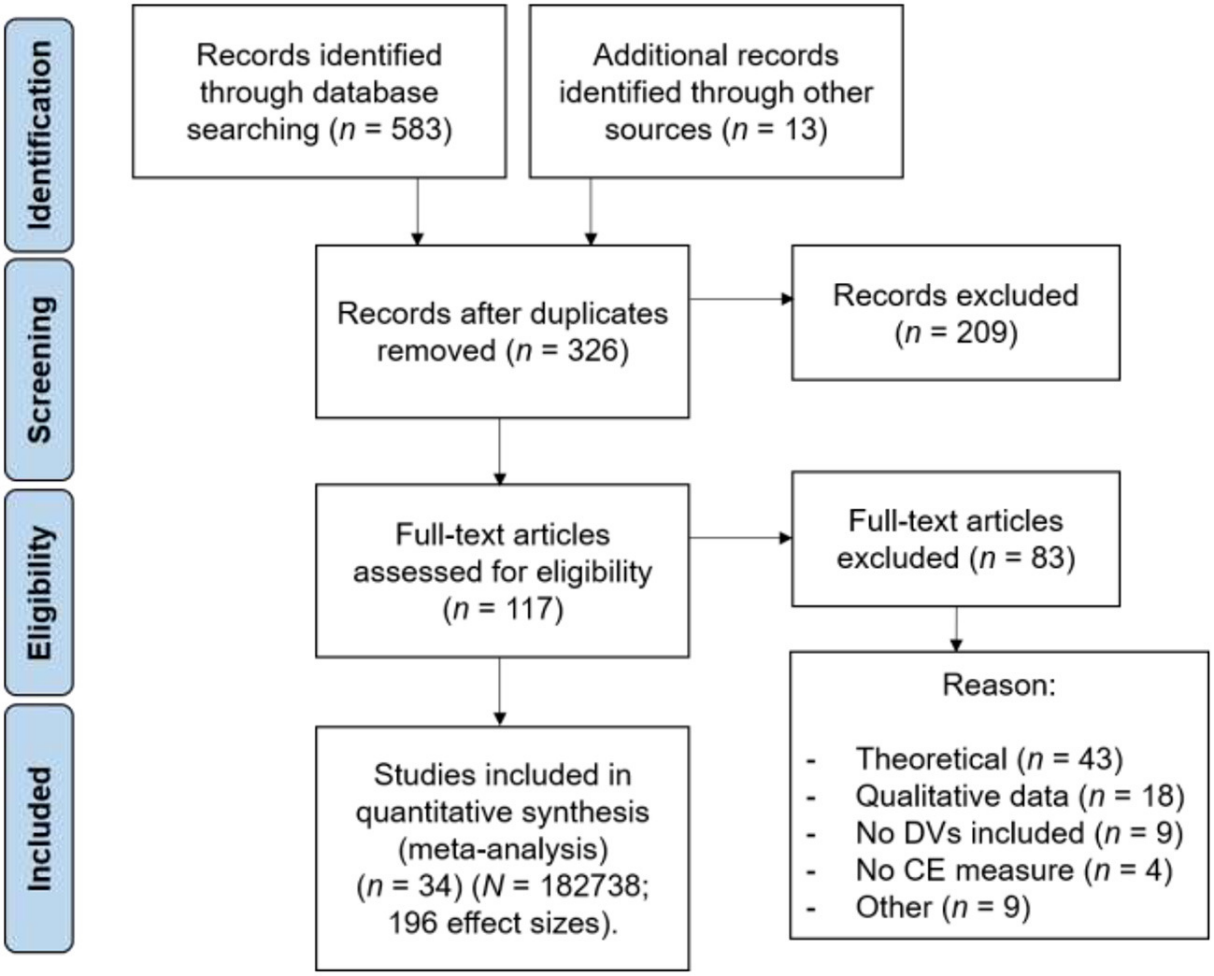
Flow chart of the studies identified and selected, following the PRISMA guidelines.

We considered studies eligible for inclusion published studies at the moment of conducting the analyses and when they fulfilled the following criteria: studies had to include (a) quantitative measurement of CE, (b) at least one criterion variable, (c) individual-level responses or aggregated data, and (d) report at least one correlation or beta coefficient between (a) and (b) (see Figure 1).

The final selection comprised 41 articles encompassing 50 studies that have included in total 182,738 participants (*Mage* = 30.8, *SD* = 9.15; 58.8% female) (see Table 1). These studies covered a wide variety of collective events: community celebrations (*N* = 10), demonstrations (*N* = 15), religious events (*N* = 10), sports gatherings (*N* = 4), music festivals (*N* = 3), others (e.g., recalling an experience of mass event; *N* = 12). The studies involved various designs (cross-sectional and longitudinal), types of collective gatherings (religious events, celebrations, and demonstrations), and measurements of collective effervescence [positive emotional intensity; PES; Tendency for Effervescence Assembly Measure (TEAM); among others; see below].

**Table 1:** Summary of approaches of empirical research on collective effervescence.

			**Attributes of the scales**		
**Theoretical background**	**References**	**Items (examples)**	**Common emotional experience**	**Valid for positive and negative affect**	**Valid for religious and secular rituals and gatherings**
Collective gatherings and *Homo duplex* and related to sacred or transcendent beliefs and values.	Moscovici, 1988/1993; Gabriel et al., 2017, 2020	“I felt as if almost everyone there felt the same emotions” “I felt as if there was a greater purpose to the event”.	X		X
CE as perceived emotional synchrony.	Páez et al., 2015; Wlodarczyk et al., 2020	“We performed as one, like a single person” “We felt stronger emotions than those we normally feel”	X	X	X
CE as emotional entrainment.	von Scheve et al., 2014; Ismer et al., 2017	“How emotionally interested have you been in…?”, “How much you have been carried away by the mood of other fans?”	X		X
CE as intense positive emotions related to social identification.	Novelli et al., 2013; Hopkins et al., 2016	“In the period of pilgrimage, to what extent have you felt alive” “I felt joyful during the demonstration”	X		X
CE related to feeling self-transcendent emotions.	Draper, 2014	“I felt awe, moral inspiration, moved by love of others/closeness or kama muta during the demonstration” “participants felt awe, inspiration and/or a sense of God's presence during religious ceremonies”	X		X

CE, Collective Effervescence. An “X” indicates whether the approach/scale has the indicated attribute for measurement.

#### Coding of the studies

Following Lipsey and Wilson's (2001) guidelines, we elaborated a coding scheme to register key information of the selected articles. It was used to record authors' names, years of publication, sample size, study design, measurement of collective effervescence, type of collective event, measures of dependent variables, and effect sizes (Supplementary Table S1). Moderators were also coded as categorical variables, and this task was performed by two investigators independently. The moderators were independently categorized by three judges with a total agreement index of 98%. Disagreements were discussed among the three judges until a consensus was reached.

#### Measurement of collective effervescence

One widely-used measure of CE consisted of scales comprising from 5 to 18 items inspired by the concept of PES described earlier in this article. These items assess mutual entrainment of intense emotions, their coordination and sharing with others, and feelings of unity with others (e.g., “We felt that we were one”, “We felt more sensitive to emotions and feelings that others feel”; Páez et al., 2015; Pelletier, 2018; Bouchat et al., 2020; Wlodarczyk et al., 2020). Another frequently used measure assessed the level intensity of affective experience or positive emotions and is generally associated with SIP-based studies, for instance, “My experiences in the crowd at the …. demonstration have been emotionally intense” (Neville and Reicher, 2011), “In the period of pilgrimage, to what extent have you felt fulfilled, happy, alive and so on?” (Hopkins et al., 2016). Other measures of effervescence focused solely on emotional entrainment (e.g., “How emotional have you felt about the…?”, “How much have you let yourself be carried away by the mood of other fans?”) (von Scheve et al., 2017), or on a combined connection with others and to the sacred (e.g., “I felt connected to others who were present at the event”, “I felt as if almost everyone there felt the same emotions”) (Gabriel et al., 2020). In the latter case, we only considered items assessing the connection with others. All information can be seen in Table 2.

**Table 2:** Descriptions and characteristics of studies included in the meta-analysis.

**ID**	**References**	**Description**	***N***	**Age *M* (*SD*)**	**% of women**	**Collective event type**	**CE scale**
S1	Alnabulsi et al., 2020	Examination of the emotional effects of participating in the *Hajj* (an annual Muslim pilgrimage to Mecca). The cross-sectional study was conducted using a convenience sample.	1,176	–	35.5	Religious event	Intensity of Positive Emotional Experience
S2	Bouchat et al., 2020	Examination of short- and long-term psychosocial consequences of participation in a major scouting event in Belgium, in 2018. The study was conducted with a convenience sample using a longitudinal design.	313	23.0 (7.5)	53.7	Community celebration	PES
S3	Carlton-Ford, 1992, S1	Study of a sample of 44 different urban communes from 7 large US cities, and their group rituals (*k* = 15) without the presence of a charismatic leader (e.g., group meditation, yoga, praying and singing). It used a convenience and hierarchical sample with a cross-sectional design.	142	–	–	Religious events	Involvement in Collective Effervescence (ICE)
S4	Carlton-Ford, 1992, S2	US urban commune rituals (see description of S5) with the presence of a charismatic leader.	144	–	–	Religious events	Involvement in Collective Effervescence (ICE)
S5	Castro-Abril et al., 2021, S1.1	Study exploring participation in the political demonstrations and social movements that started in Chile in October 2019. It was conducted with a convenience sample of participants using a cross-sectional design.	186	34.8 (12.1)	65.5	Demonstration	PES
S6	Castro-Abril et al., 2021, S1.2	Exploration of Chilean social movements (see description of S7). This study used a convenience sample of spectators who followed the movements live or in a mediated fashion (e.g., online, on TV).	65	38.5 (12.7)	65.7	Demonstration	PES
S7	Corcoran, 2015	Cross-sectional study using data (aggregated level) from the 2001 US Congregational Life Survey (USCLS), which analyzed 344 religious congregations (e.g., Pentecostal, Black Protestant and Catholic) and attendees at collective religious rituals. Hyper-network sampling was used to gather a random sample of congregations.	46,571	48.5 (15.2)	–	Religious event	Perception of CE
S8	Corcoran, 2020	Cross-sectional study conducted with the 2001 US Congregational Life Survey (USCLS) (see S9).	49,360	48.80 (15.35)	60.1	Religious event	Emotional Energy Index
S9	Cusi et al., 2022	Cross-sectional study that assesses (through recall of a past experience) participation in past collective events. Specifically, the type of event (e.g., family reunions, concerts, etc.) and the frequency of participation are evaluated.	372	23.36 (6.85)	67.2	Other type	PES
S10	Draper, 2014	Study using data (aggregated level) from the 2001 US Congregational Life Survey (USCLS) (see S9). This study used a cross-sectional design.	73,196	–	62.0	Religious event	CE Index
S11	Drengner et al., 2012	Study exploring participation in Europe's biggest hip-hop festival in Germany. Mainly designed as a music festival, it also includes different aspects of hip-hop culture (e.g., graffiti, breakdancing) and is attended by up to 20000 visitors. The study was carried out using a cross-sectional design with a convenience sample.	409	21.5 (3.1)	33.0	Music festival	Intensity of Positive Emotional Experience
S12	Fischer et al., 2014	Measurement of quantified physiological fluctuations (heart rates) and self-reported affective states at the *Thimithi* festival in a Hindi community in Mauritius. The 10-day festival ends with a procession and subsequent fire-walking ritual. The final sample included fire-walkers (of whom 13 participated in body piercing) and spectators who were evaluated pre- and post-event.	70	32.6 (14.9)	49.0	Religious event	Involvement in the Ritual
S13	Gabriel et al., 2017, S1	Cross-sectional study using the Tendency for Effervescent Assembly Measure (TEAM scale) with an undergraduate student sample (University at Buffalo, US).	117	19.0 (3.4)	53.0	Other type	TEAM
S14	Gabriel et al., 2017, S2	Exploration of the Tendency for Effervescent Assembly Measure (TEAM; see S16). This study included data from a second undergraduate student sample.	163	18.9 (1.4)	52.8	Other type	TEAM
S15	Gabriel et al., 2017, S3	Evaluation of the Tendency for Effervescent Assembly Measure (TEAM; see S16). This study included data from a community sample.	405	35.4 (12.4)	43.7	Other type	TEAM
S16	Gabriel et al., 2017, S5	Study evaluating past experiences of collective effervescence with an undergraduate student sample from the University at Buffalo (US). It explores the role of social needs fulfillment in effervescent assembly, as well as the relationship of the scale with recent collective effervescence experiences using a cross-sectional design.	150	19.4 (5.3)	52.6	Other type	TEAM
S17	Gabriel et al., 2020, S3a	In this study, recruited participants (university students from a large US city) recalled recent collective effervescence experiences in a large crowd of people. The design used was cross-sectional.	273	19.0 (1.2)	33.3	Other type	State Collective Effervescence
S18	Gabriel et al., 2020, S3b	Cross-sectional study measuring previous experiences in a big crowd during some kind of gathering. Participants were recruited through a US online site.	239	51.2 (17.6)	74.0	Other type	State Collective Effervescence
S19	Hopkins et al., 2016	Study conducted on the *Magh Mela* pilgrimage (annual event that attracts millions of pilgrims to the banks of the Ganges at Prayag). Many participants (known as *kalpwasis*) commit to staying for a full month and to participating for 12 consecutive years, and subject themselves to a distinctive routine of religious devotion (e.g., bathing in the Ganges, praying). The study was carried out with a convenience sample of *kalpwasis* using a cross-sectional design.	416	64.4 (9.3)	57.0	Religious event	Intensity of Positive Emotional Experience
S20	Jiménez et al., 2005	Longitudinal study evaluating emotional mechanisms (e.g., social sharing) in the context of demonstrations against terrorism following the 2004 Madrid train bombings (11-M). The study was carried out with a convenience sample of university students from 8 Spanish universities and their acquaintances.	675	27.6 (11.7)	71.0	Demonstration	Intensity of Positive Emotional Experience
S21	Kettner et al., 2021	Longitudinal study evaluating perceived emotional synchrony during psychedelic rituals and prediction of fusion of identity, psychological wellbeing and social connectedness 4 weeks after.	495	44.3 (12.2)	44.0	Other type	PES
S22	Naidu et al., 2022, S2	Recollection of past online experiences of collective effervescence. Participants were instructed on different types of experiences in the context of the COVID-19 pandemic and were asked to describe one.	353	19.27 (1.6)	43.3	Other type	Collective Effervescence Experiences
S23	Neville and Reicher, 2011, S3	Study exploring the experience of participation in the three-day Rock Ness festival (2009, UK), an event held on an annual basis (until 2013) featuring a mixture of rock and dance acts that was attended by approx. 30000 participants. The study was carried out with a convenience sample using a cross-sectional design.	98	26.6 (–)	49.0	Music festival	Intensity of Positive Emotional Experience
S24	Novelli et al., 2013, S1	Cross-sectional exploration of the effects of participation in a free outdoor music event featuring DJ Fatboy Slim, in 2002 (Brighton, UK). It was a very crowded event (*N* ≈ 250,000) and respondents (convenience sample) received £5 for participating.	48	35.9 (7.5)	67.0	Music festival	Intensity of Positive Emotional Experience
S25	Páez et al., 2013	Studying exploring demonstrations in the context of an important large-scale social protest movement in Spain during May 2011 (also known as the 15-M movement), triggered by declining economic and social conditions. The convenience sample included participants in several cities (e.g., Madrid, Barcelona) and the design used was cross-sectional.	213	29.4 (11.8)	55.6	Demonstration	PES
S26	Páez et al., 2015, S1	Cross-sectional study evaluating participation in annual pseudo-military folkloric marches in Belgium. This ritual includes dressing up in historical military uniforms and bearing old weapons while marching in synchrony. Participants were recruited at a rehearsal meeting and were all from the same town.	93	32.6 (12.9)	19.4	Community celebration	PES
S27	Páez et al., 2015, S4.1	Study of an experimentally-induced demonstration in which participants (university students from the UPV/EHU, Spain) were asked to create banners with antiracist slogans in support of a local NGO (*SOS Racism*). The data focus on the experimental condition, i.e., the collective creation of slogans, and the study used a longitudinal design.	35	21.7 (4.1)	91.4	Demonstration	PES
S28	Páez et al., 2015, S4.2	Study of an experimentally-induced demonstration (see S30). This study includes data from participants in the control condition (i.e., individual banner creation in the presence of others) and used a longitudinal design.	40	20.7 (1.2)	82.5	Demonstration	PES
S29	Parveen and Khan, 2020	Correlational study assessing the participation of religious devotees to a visit to Banner Sharif and Piran Kalyar mausoleums.	100	–	39	Other type	PES
S30	Pelletier, 2018	Belgian citizens were recruited through a probability sampling procedure in Bourse Square in Brussels (Belgium), during the spontaneous collective gatherings that followed the March 22 (2016) terrorist attacks. The study used a cross-sectional design.	198	34.9 (15.2)	49.5	Demonstration	PES
S31	Pizarro et al., 2017, S1.1	Study of an experimentally-induced collective demonstration in favor of immigrants from the Maghreb (also known as Northwest Africa), supported by a local NGO (*SOS Racismo*). The sample comprised university students studying Social Work (UPV/EHU, Spain) divided into different conditions in accordance with the information they were given to create the banners and subsequently engage in the demonstration. This study focuses on the first experimental condition, which used exclusively human information (i.e., traits and characteristics that are uniquely human) and had a longitudinal design.	24	20.0 (1.3)	70.8	Demonstration	PES
S32	Pizarro et al., 2017, S1.2	Study of an experimentally-induced collective demonstration (see S34). This study included participants randomized to the second experimental condition, using non-exclusively human information (i.e., traits and characteristics that are shared with other animal species) to create the banners.	30	21.9 (6.7)	80.0	Demonstration	PES
S33	Pizarro et al., 2017, S1.3	Study of an experimentally-induced collective demonstration (see S34). This study included participants randomized to the control condition, using utilitarian information (i.e., information centered on the economic gains of receiving immigrants) to create the banners.	29	20.2 (1.8)	79.3	Demonstration	PES
S34	Pizarro et al., 2020	Quasi-experiment centered on the effects of a mindful dancing program lasting 45 min, consisting of a guided mindfulness meditation carried out while performing a series of synchronous movements, guided by a professional. Participants were university students (UPV/EHU, Spain) and this study focuses on the intervention group, using a longitudinal design.	67	20.3 (1.9)	82.1	Sports gathering	PES
S35	Pizarro et al., 2021, S1.1	Using a cross-sectional design, this study evaluates the effects of past participation in collective rituals and gatherings (recall approach) on global identity and prosocial intentions. This study was carried out with a convenience sample of participants from Mexico.	373	23.4 (6.9)	68.1	Other type	PES
S36	Pizarro et al., 2021, S1.2	Study evaluating past participation in collective rituals and gatherings, with a sample of participants from Mexico and the Basque Country (Spain).	145	27.9 (10.5)	64.1	Other type	PES
S37	von Scheve et al., 2014	Naturalistic study of participation in the 2010 Football World Cup that evaluates the effects of emotional entrainment and collective emotions. It used a longitudinal design with a convenience sample.	98	28.4 (11.4)	37.0	Sports gathering	Experience of Emotional Entrainment
S38	von Scheve et al., 2017	This study measures the effects of participation in a mega-sporting event (the UEFA championship) in 2012 and includes participants from Germany (*n* = 302), the UK (*n* = 144) and Poland (*n* = 61). It used a longitudinal design with convenience samples.	507	37.22 (13.89); 45.28 (15.02); 28.28 (8.37)	55.0; 48.6; 59.0	Sports gathering	Experience of Emotional Entrainment
S39	Wlodarczyk et al., 2020, S1	Study conducted in the context of the *Tamborrada*, an annual ritual held in Donostia-San Sebastián (northern Spain), which involves large groups of drummers who invade the city for a 24-hour-long celebration. The groups march and play folk songs in exact or complementary synchrony and costumes include barrel-holders, cooks, and Napoleonic-style military personnel. The study was carried out with a convenience sample using a longitudinal design.	550	42.7 (13.9)	47.8	Community celebration	PES
S40	Wlodarczyk et al., 2020, S2.1	Study of participation in a patriotic paramilitary parade held annually in Chile (May 21 Iquique Naval Combat). Data were gathered from high-school students who participated in a synchronous march accompanied by marching bands. The study was carried out with a convenience sample using a longitudinal design.	151	16.4 (16.4)	37.7	Demonstration	PES
S41	Wlodarczyk et al., 2020, S2.2	Study of the effect of participating in newcomer hazing rituals on the University of Louvain campus (Belgium). This tradition involves enacting costly rituals (e.g., disgusting stimuli, humiliations) with first-year students, and is frequently practiced in different sororities and fraternities, etc. The study used a longitudinal design with a convenience sample.	120	19.5 (3.0)	74.0	Community celebration	PES
S42	Wlodarczyk, Zumeta et al., 2021	Longitudinal study comparing participants in Sunday Mass with participants in secular Sunday group activities (e.g., family lunch, sporting activities). The study was carried out with a convenience sample.	110	53.9 (18.2)	61.8	Religious event	PES
S43	Xygalatas et al., 2013	Cross-sectional study of the effects of two rituals which form part of *Thaipusam*, an important religious Hindu festival in Mauritius. One of the rituals consisted of singing and collective prayer, and the other of body piercing and other painful actions. All participants took part in both rituals and were randomized to be tested in only one.	86	32.6 (14.9)	49.0	Religious event	Involvement in the Ritual
S44	Zlobina and Celeste, 2022, S1	Correlational study studying participation in applause rituals (i.e., collective displays of gratitude directed at healthcare personnel working in the COVID-19 pandemic in Spain) during confinement.	528	42.85 (14.55)	69	Demonstration	PES
S45	Zlobina and Celeste, 2022, S2	Correlational study of the participation in an applause ritual (see S53).	292	21.13 (2.45)	78	Demonstration	PES
S46	Zumeta et al., 2016	Study evaluating the effects of engaging in different collectively-performed physical and sporting activities (e.g., football, volleyball, aerobics, dancing, hiking, etc.). It used a recall of event approach with a convenience sample and a cross-sectional design.	276	21.6 (4.1)	72.0	Sports gathering	PES
S47	Zumeta et al., 2020	Cross-sectional study in the context of marches for women's rights in 9 countries. The demonstrations were mass gatherings during the 8th of March, 2019 and the participants were recruited through convenience samples.	2,843	30.55 (11.66)	83.8	Demonstration	PES
S48	Zumeta et al., 2020, S1	Study exploring the effects of participating in *Bizilagunak*, an intercultural family lunch promoted by a local NGO (*SOS Racism*). The event consisted of more than 200 meals occurring simultaneously, in which participants were divided into hosts and attendees with the intention of promoting interaction between Basque natives and immigrants. The study used a longitudinal approach and a convenience sample.	196	38.1 (13.1)	75.5	Community celebration	PES
S49	Zumeta et al., 2020, S2	Study of a communal celebration entitled Rices of the World, consisting of a community lunch held in a public area using rice as the common thread linking different cultures. The study was carried out with a convenience sample and used a cross-sectional design.	107	37.8 (12.7)	50.5	Community celebration	PES
S50	Zumeta et al., 2020, S3	Cross-sectional study on the 16th and 17th demonstrations against racism and xenophobia, held in Donostia-San Sebastián (Spain). These marches are held annually and are attended by between 600 and 700 people. The study was carried out using a convenience sample.	91	45.8 (11.5)	52.7	Demonstration	PES

ref. indicates the study reference. *M*(*SD*) indicates the mean and standard deviation, respectively. An uppercase “S” followed by a number indicates the study as it is presented in the corresponding article. Dashes (–) indicate that the information is not reported. CE, Collective Effervescence; PES, Perceived Emotional Synchrony; TEAM, Tendency for Effervescent Assembly Measure.

#### Criterion variables

Our list of criterion variables is the result of a compromise between the outcomes of collective events that have emerged from our literature review and the variables available in the studies included in this meta-analysis. We have organized these variables into two categories, according to a time- and scope-related approach thus, creating proximal and distal outcomes. The list of variables are thus organized in (1) individual emotions felt by participants, (2) communal sharing or immediate social integration, (3) collective emotions, (4) social integration, (5) social values and beliefs, and (6) empowerment. We made a distinction between immediate effects (i.e., what happens during the collective gathering itself) and more or less long term effects (i.e., what individuals or publics retain from their participation in the demonstration, collective ritual, etc.). In addition, immediate effects represent facets or features that conform the construct of CE.

##### Proximal outcomes

**Figure 2:**
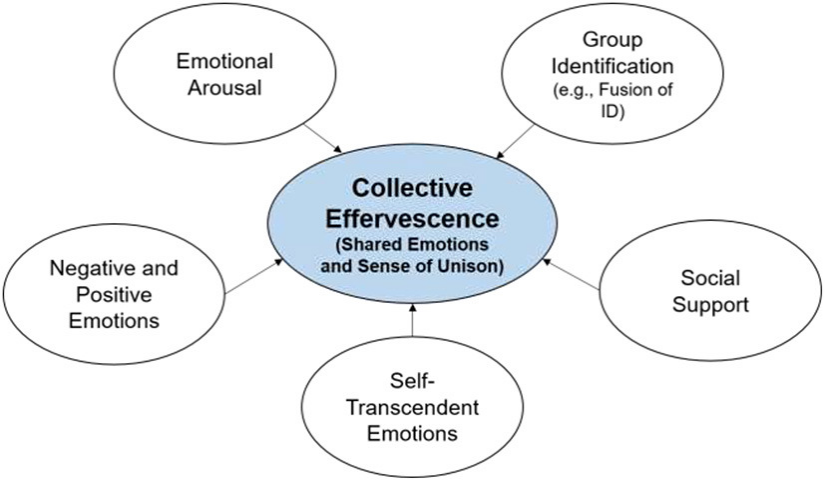
Proximal outcomes-construct validity criteria of collective effervescence.

In this category, we included variables addressing either the participants' own states and feelings, or how they feel with regard to co-present participants. Such effects can therefore directly affect the course of the collective situation namely, by intensifying emotional arousal and positive emotions or by feeding up an experience of self-transcendence. Other proximal variables are related to situated or immediate social integration, like altering the perception of individual boundaries, enhancing openness to others. In all, these variables also serve as construct validity criteria, since they are constructs that cover the two essential elements of CE found in the literature review: emotional reactions and a sense of union with others. The variables included here (i.e., emotional arousal, positive emotions and self-transcendent emotions, and a sense of unison with others) are considered by scholars as criteria or indexes of a state of CE (Draper, 2014; von Scheve et al., 2014; Páez et al., 2015; Hopkins et al., 2016; Fiske et al., 2017; Gabriel et al., 2020; Wlodarczyk et al., 2020). Variables considered in this category are as follows (see Figure 2).

###### Individual emotions

This class comprised self-reported individually-felt emotional states (e.g., DESm, Fredrickson, 2009) including negative emotions (e.g., “How sad, discouraged, or unhappy have you felt?”), positive emotions (e.g., “What was the most joyful, glad, or happy you felt?”), and self-transcendent positive emotions (e.g., “What is the most inspired, uplifted, or elevated you felt during the event?”) related to participation in the collective gathering. When possible, we additionally computed an indicator of general arousal (i.e., averaging absolute values of positive and negative emotions).

###### Communal sharing or immediate social integration

This class gathered variables assessing the activation of the communal sharing mode (i.e., intensification of horizontal relationships, see Fiske, 1992). It included indexes of self-categorization as a member of the group, or feeling that the individual and collectives selves overlap or merge. For instance, activation of a proximal social identity (e.g., “It is nice to be part of my group”) (e.g., Leach et al., 2008), verbal (e.g.; “I am one with my group”) and pictorial expressions of identity fusion (Swann et al., 2009; Gómez et al., 2011, respectively), and the perception of support (Social Support-related scales like Richer and Vallerand's (1998); see (Drury et al., 2016); e.g., “If I need help, other pilgrims would help me”). Of particular importance, we only considered cases where the identification explicitly referred to co-participants in the collective event.

##### Distal outcomes

**Figure 3:**
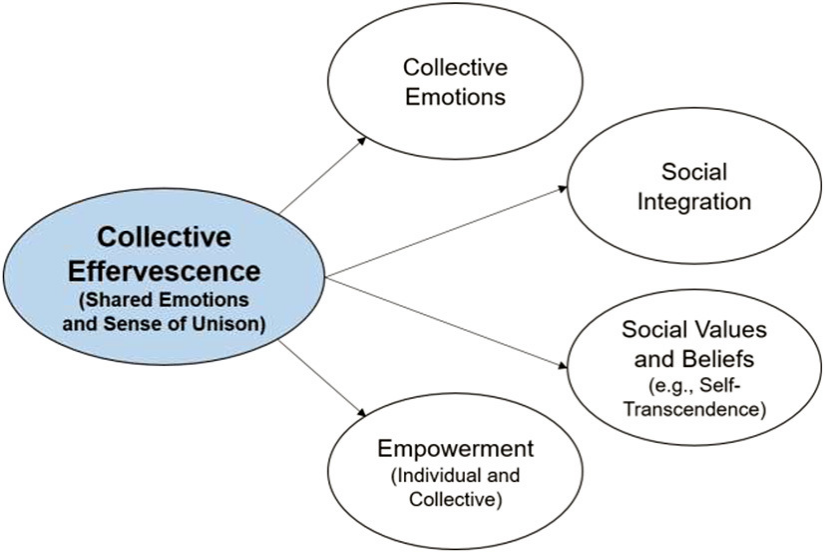
Distal outcomes of collective effervescence.

This second class of outcome variables gathered effects that extend beyond the collective situation itself. Some of these variables were assessed immediately after the collective situation but their target extends beyond this situation (e.g., unison felt beyond co-present participants—with the whole community, or with a broad social movement; e.g., a positive emotional atmosphere felt beyond the punctual climate). Other distal outcome variables were assessed in follow-up measurements and thus regarded effects that participants carry with them in the period after the collective event. The following variables were considered under this second category (see Figure 3).

###### Collective emotions

Several studies included a Perceived Emotional Climate scale assessing the emotional atmosphere as currently perceived by respondents [i.e., CEP-N or CD-24, by de Rivera and Páez, 2007; e.g., “The general mood or social climate is: (a) Hopeful, (b) anger, hostility, aggressiveness among people”]. This scale involves two dimensions: negative perceived emotional climate and positive perceived emotional climate. Therefore, it provides indicators of what conceptually represents the shared moods and emotions of a group—or collective emotions (see von Scheve and Salmela, 2014).

###### Social integration

Variables included in this class assessed the extent to which participants commit to the group or event (e.g., “I intend to visit [name of the event] in the future”, as in Drengner et al., 2012) or identify with an extended group (e.g., “Do you have a strong sense of belonging to this congregation”, as in Draper, 2014; or a sense of ingroup solidarity using the city as a target, as in Pizarro, 2019). In all cases, the measures considered in this class were not strictly targeted at co-participants in the collective gatherings.

###### Social values and beliefs

This class included Self-Transcendent Beliefs [e.g., “I have had moments of great joy in having strong feelings of unity”] of Cloninger et al. (1994) scale, as in Zumeta et al. (2016) and Values (e.g., “It's very important to her to help the people around her. She wants to care for their wellbeing”, from Schwartz, 2007). The class also comprised other forms of self-transcendent beliefs such as Purpose in Life (e.g., Meaning in Life Questionnaire, used in Gabriel et al., 2017) and Spirituality [Piedmont's ASPIRES scale (Piedmont, 2004), used in Pizarro et al., 2021].

###### Empowerment

This class included a measure of perceived vitality (e.g., Ware and Sherbourne's, 1992 SF-36, used in Zumeta et al., 2016) and variables tapping a sense of empowerment measured at both individual and collective levels. The latter comprised measures of wellbeing (e.g., Satisfaction with Life Scale, as in Gabriel et al., 2020; or Pemberton's Happiness Index, as in Pizarro et al., 2017), Self-esteem (e.g., Rosenberg's individual self-esteem (Rosenberg, 1965); e.g., “Overall, I am satisfied with myself”; Luthanen and Crocker's collective self-esteem, 1992; e.g., “I am a worthy member of the social groups I belong to”), and of Collective Efficacy (van Zomeren et al., 2010; e.g., “I believe that together we can change the current situation” or “We realized we were perfectly capable of achieving our aims”).

#### Moderators

In a subsequent set of analyses, we examined the effects of collective effervescence on the criterion variables across several potential moderators. We only conducted moderation analyses when there were at least two levels of the moderator with a representation of at least *k* = 3. The present article only includes the moderation of type of event, but all analyses can be seen on Supplementary material online.

##### Type of collective gathering or event

Effect sizes were therefore compared for studies featuring demonstrations (i.e., high in negative emotions = 1), celebrations (i.e., high in positive emotions = 2) and religious events (=3). This distinction allows a more-grained exploration of effects depending on the actual content of gatherings.

##### Study design

Effect sizes observed for cross-sectional (1) and longitudinal (2) designs were compared. Significantly larger effect size in longitudinal studies can strengthen its interpretation in terms of causal link.

##### Type of CE scale

This moderator took into consideration the distinction between the different types of measurements of collective effervescence mentioned above. Thus, a distinction was made between collective effervescence measured (1) as PES (*k* = 28), (2) as an experience of intense and positive affect (*k* = 6), and (3) using other scales that focus on mutual emotional entrainment or connection with others (*k* = 16)—the limited number of studies identified prevented us from differentiating between these last two. In addition, we compared the effect sizes for the short (1) and long (2) forms of the PES scale, expecting that the short form would prove as valid as the long one.

##### Demographics and cultural values

Finally, we explored the possible moderating effects of age, gender, on the one side, and national levels of Power Distance Index and Individualism-Collectivism, on the other. The latter, were conducted using the national level (i.e., using the country where the ritual was enacted) value of Hofstede's (2015) cultural dimensions, as in Agostini and van Zomeren (2021).

#### Data analyses

The analyses were carried out using R (version 4.0.4) (R Core Team, 2020) and the *metafor* package (version 3.0.2) (Viechtbauer, 2021). We applied random-effects models to fit the relations between CE and the criterion variables, following the guidelines proposed by Rosenthal (1979), Hunter and Schmidt (2004) and Cumming (2013).

##### Effect sizes and correction for attenuation

We used Pearson's *r* as a measure of effect size given both its simplicity and the fact that this statistic is commonly employed in most studies. When correlations were not available—neither in the full text nor in subsequent requests to authors—, we computed them from regression coefficients according to Peterson and Brown's (2005) instructions. However, and since this procedure tends to inflate correlations, we deliberately removed those larger than 0.90. Since some studies could report several effect sizes for a single construct, some effects are likely nested and thus, independence assumptions of the observations cannot be met (Lipsey and Wilson, 2001). For this reason, such a dependency problem needs to be compensated for to reduce the bias of estimates. We dealt with this problem by including only one selected effect size from each study, for a given dependent variable.

When the reliability index of the CEe scale and a given criterion measure were known for each study, then the individual effect sizes can be corrected for attenuation due to unreliability before conducting the meta-analysis. This was conducted by dividing direct *r*s by the square root product of Cronbach's alphas of the two measures (i.e., rho values). These provide an estimation of the effect sizes corrected by the reliability of the scales used (see Hunter and Schmidt, 2004) and we used them to accompany all main results.

##### Publication bias and robustness of effects

Publication bias refers to the fact that studies with statistically significant effects are more likely to be published than studies with null effects, meaning that the published literature will be skewed toward positive effects, which will in turn bias meta-analyses. We explored this through a series of analyses. We did not include a funnel plot test in our study, as funnel plots do not provide valid estimates of publication bias when fewer than 30 studies are included (Lau et al., 2006). In this study, we employed rank correlation test (Begg and Mazumdar, 1994) and the regression test (Sterne and Egger, 2005) which use the standard error of observed outcomes as predictor to check funnel plot asymmetry. We considered absence of publication bias when these two tests are non-significant; possible publication bias when at least one is significant, and a high possibility of publication bias when both are significant. In addition, we conducted an examination of the studentized residuals and Cook's distances (Cook and Weisberg, 1982). These analyses provide empirical tests to explore whether any given study should be considered an outlier (i.e., studentized residual larger than ±2.914) or overly influential (i.e., a proportion of Cook's distance and *k*, calculated with and without a given observation). We considered that a given study might be influential when any of these two conditions are fulfilled and a high possibility when these two criteria are met (for more details about possibly influential studies, see Viechtbauer, 2021).

Regarding robustness of the effects, we used fail-safe *N* tests (Rosenthal, 1979) (see Rubio-Aparicio et al., 2018) which represent how many new—or missing—studies with a zero-effect size would be needed to transform a significant *p*-value into a non-significant one. Should it emerge that only a few studies—say five or ten—were necessary to “nullify” the effect, then we would be concerned that the true effect was indeed zero (Borenstein et al., 2009). Rosenthal (1979) suggested a fail-safe *N* value above 5; *k* + 10 reflects results that are tolerant to contradicting studies, where *k* is the number of studies included in the meta-analysis. Rosenthal noted this is a conservative threshold, meaning that if the fail-safe *N* is well above this value, there is increased confidence that the observed effect size estimate is trustworthy.

##### Heterogeneity analyses

To analyze possible heterogeneity, we used several indicators including the *Q* test of heterogeneity (Cochran, 1954) the *I*^2^ statistic (Higgins and Thompson, 2002) and the τ^2^. The *Q* test evaluates whether the distribution of effect sizes around the mean is broader than predicted based on sampling error alone (i.e., presence-absence of heterogeneity), and thus it suggests that a random model is more suitable. The *I*^2^ statistic, on the other hand, describes the percentage of variation across studies that is due to heterogeneity rather than change (i.e., percentage of real variability). Finally, τ^2^ along its standard error, indicates absolute value of the true variance (i.e., heterogeneity) and is considered as the real importance of variability since it presents the value in terms of the scale of the effect size.

##### Comparison of reported effects

Finally, in order to establish comparable criteria of the reported effects, we adopted the following standards: effects of up to *r* < 0.18 were considered small, effects of *r* = 0.18–0.32 were considered medium and *r* > 0.32 was considered indicative of a large effect. These criteria were adopted because they avoid the limitations faced by Cohen's (1977) qualitative guidelines (see Hunter and Schmidt, 2004; Funder and Ozer, 2019; Correll et al., 2020). (Gignac and Szodorai, 2016) found that a low or lower quartile effect is *r* = 0.11 or less, between 0.12 and 0.19 is a lower-middle quartile, between 0.20 and 0.29 is an upper-middle quartile and above 0.29 is high. The equivalents for the correlation corrected for attenuation or measurement error (i.e., the real correlation) were, respectively, rho = 0.16 or less, 0.17–0.25, 0.26–0.37, and 0.38 or more (Lipsey and Wilson, 2001; Gignac and Szodorai, 2016). Overall, they are considered more realistic according to meta-analytical reviews.

## Results

### Associations with proximal or construct validity outcomes

#### Individual emotions

As displayed in Table 3, CE was significantly associated with individual emotional activation, regardless of emotional valence. The *Q* test was significant and the *I*^2^ squared shows that the percentage of variation between studies due to heterogeneity is significant, presenting 95.42% (above the mean of 71–74% that is common in meta-analyses, see Stanley et al., 2018). The randomized model, better suited by the high heterogeneity, and by the fact that the studies have been conducted in different countries, shows a significant effect [*r* = 0.44, 95% CI (0.32, 0.56)].^2^ This effect is high, above the median of social and organizational psychology studies (i.e., *r* = 0.18 and 0.16), and included in the fourth or highest quartile (Richard et al., 2003; Bosco et al., 2015; Gignac and Szodorai, 2016). Rosenthal's fail-safe *N* analysis (*N*_fs_ = 22,003) brought a much robust result and neither the rank correlation nor the regression test indicated any funnel plot asymmetry (*p* = 0.914 and *p* = 0.702, respectively).

**Table 3:** Pooled correlations between collective effervescence and criterion variables.

**Criterion Variables**				**Effect sizes**		**Heterogeneity**			**(80% CI Pred. Intv.)**
**Dimension**	**Variable**	***k***	***N***	***r* (95% CI)**	**rho (95% CI)**	***Q*(*df*)**	***I* ^2^**	**τ^2^ (SE)**	
**Proximal outcomes**								
Individual emotions	Arousal	14	48,316	0.443 (0.322, 0.564)	0.506 (0.367, 0.646)	*Q*(13) = 180.020***	95.42	0.046 (0.020)	(0.158, 728)
	Negative emotions	14	2,028	0.047 (−0.053, 0.147)	0.056 (−0.057, 0.168)	*Q*(13) = 71.198***	78.23	0.025 (0.013)	(−0.166, 260)
	Positive emotions	22	5,834	0.547 (0.468, 0.625)	0.608 (0.526, 0.690)	*Q*(21) = 239.707***	94.30	0.030 (0.011)	(0.318, 0.775)
	ST emotions	17	5,340	0.577 (0.500, 0.653)	0.641 (0.559, 0.723)	*Q*(16) = 171.312***	92.90	0.021 (0.009)	(0.385, 0.769)
Communal sharing	Ingroup ID	14	3,253	0.456 (0.351, 0.562)	0.498 (0.389, 0.608)	*Q*(13) = 302.072***	92.69	0.034 (0.015)	(0.211, 0.702)
	FI Verbal	5	1,031	0.694 (0.660, 0.729)	0.734 (0.686, 0.781)	*Q*(4) = 6.958	6.94	0.000 (0.001)	(0.668, 0.721)
	FI Pictorial	11	1,504	0.347 (0.250, 0.444)	0.364 (0.262, 0.466)	*Q*(10) = 48.937***	71.66	0.016 (0.011)	(0.173, 0.521)
	Social Support	12	4,135	0.334 (0.247, 0.421)	0.376 (0.278, 0.473)	*Q*(11) = 181.691***	89.64	0.020 (0.010)	(0.143, 0.526)
**Distal outcomes**								
Collective emotions	Negative climate	5	1,357	0.017 (−0.105, 0.138)	0.026 (−0.118, 0.170)	*Q*(4) = 19.967***	77.81	0.015 (0.012)	(−0.157, 0.190)
	Positive climate	4	1,159	0.248 (0.089, 0.406)	0.328 (0.095, 0.562)	*Q*(3) = 25.025***	86.13	0.022 (0.019)	(0.030, 0.465)
Social integration	Ingroup commitment	7	123,962	0.372 (0.288, 0.456)	0.418 (0.326, 0.510)	*Q*(6) = 1484.496***	99.24	0.011 (0.007)	(0.229, 0.515)
	Ingroup ID (extended)	8	75,139	0.320 (0.205, 0.435)	0.368 (0.230, 0.505)	*Q*(7) = 150.888***	94.23	0.024 (0.014)	(0.106, 0.534)
Social values and beliefs	ST beliefs	5	4,231	0.435 (0.278, 0.592)	0.484 (0.319, 0.650)	*Q*(4) = 126.216***	96.51	0.030 (0.020)	(0.192, 0.678)
	ST values	4	1,103	0.335 (0.282, 0.387)	0.379 (0.329, 0.430)	*Q*(3) = 2.699	0.06	0.000 (0.002)	(0.300, 0.369)
	Purpose in life	8	4,478	0.358 (0.232, 0.484)	0.436 (0.295, 0.577)	*Q*(7) = 291.422***	94.70	0.027 (0.016)	(0.131, 0.585)
	Spirituality	5	1,416	0.374 (0.284, 0.464)	0.415 (0.315, 0.515)	*Q*(4) = 17.660***	73.38	0.007 (0.007)	(0.249, 0.499)
Empowerment	Vitality	5	1,411	0.243 (0.180, 0.305)	0.247 (0.184, 0.311)	*Q*(4) = 7.153	30.91	0.002 (0.003)	(0.178, 0.308)
	Wellbeing	17	6,188	0.316 (0.236, 0.395)	0.354 (0.264, 0.445)	*Q*(16) = 158.663***	89.84	0.021 (0.009)	(0.121, 0.510)
	Self esteem	5	829	0.154 (0.023, 0.285)	0.201 (0.027, 0.374)	*Q*(4) = 20.120**	73.42	0.016 (0.014)	(−0.030, 0.337)
	Collective efficacy	7	1,471	0.464 (0.391, 0.537)	0.508 (0.437, 0.579)	*Q*(6) = 20.594***	64.71	0.006 (0.005)	(0.357, 0.572)
	Collective self-esteem	7	1,497	0.421 (0.284, 0.558)	0.485 (0.327, 0.643)	*Q*(6) = 46.607***	90.80	0.027 (0.018)	(0.191, 0.651)

*k* is the number of studies and *N* the number of participants included in the analysis. *r* and rho (95% CI) indicate pooled Pearson's *r*s and rhos (i.e., the estimation of the effect with a correction for reliability) and their 95% confidence intervals. *Q*(*df* ) indicate *Q* heterogeneity test and its degrees of freedom. *I*^2^ indicate a percentage indicating the relationship between residual and unaccounted heterogeneity. τ^2^ and (SE) indicate the estimated amount of residual heterogeneity and its standard error. (80% CI Pred. Intv.) represents the 80% confidence intervals of prediction intervals (Riley et al., 2011). *, **, ***, indicate *p* < 0.05, 0.01, and 0.001, respectively.

CE was also significantly associated with individual Positive Emotions, *r* = 0.55, and with Self-Transcendent Emotions, *r* = 0.58. Fail-safe *N* tests returned values of 28,425 and 21,636, respectively. For the association with Positive Emotions, Egger's regression test showed a significant value (*p* = 0.016 and 0.060, for Self-Transcendent Emotions), but no the rank correlation test (*p* = 0.129 and 0.393). In the case of negative emotions, CE was not significantly associated with negative affect.

#### Communal sharing

CE was significantly and positively associated with every measure of social integration that involved a relationship between the participant and their ingroup or people participating in the collective gathering, with large effect-size correlations ranging from *r*_*pooled*_ = 0.33–0.69. Specifically, it was associated with every form of ingroup identification, namely with Ingroup Identity (*r* = 0.47) as well as with the verbal (*r* = 0.69) and pictorial measures of Fusion of Identity (*r* = 0.35). In all cases, fail-safe *N* tests (*N*_fs_s = 4,773, 2,607, and 794, respectively), and regarding asymmetry of the funnel plot, neither the rank correlation nor the regression test indicated asymmetry for Ingroup Identity (*p* = 0.233 and *p* = 0.364, respectively), for the verbal measure (*p* = 0.817 and *p* = 0.956, respectively) of for the pictorial measure of Fusion of Identity (*p* = 0.197 and *p* = 0.275, respectively).

Finally, CE was also associated with the perception of receiving social support from ingroup members, *r* = 0.33, with a large fail-safe *N* and a non-significant rank correlation nor the Egger's regression (*N*_fs_ = 2,198; *p* = 0.197, and *p* = 0.275, respectively).

### Associations with distal outcomes

#### Collective emotions

CE was associated with Positive Emotional Climate (*r* = 0.25), or the perception that people feel shared positive emotions for a given period. The fail-safe *N* test (*N*fs = 99) and a non-significant rank correlation and Egger's regression (*p* = 1.000 and *p* = 0.759, respectively) indicated robust effects and absence of publication bias. Pooled *r*s revealed that collective effervescence was not significantly associated with Negative Emotional Climate.

#### Social integration

CE correlates with self-reported participants' investment in the group (i.e., Ingroup Commitment) with *r* = 0.37, as well as with the identification with an extended ingroup *r* = 0.32. For both cases, subsequent analyses showed robust effects and excluded publication biases (*N*_fs_ = 17872; rank correlation and regression test, *p* = 0.773 and 0.171, respectively; and *N*_fs_ = 14597; rank correlation and regression test, *p* = 0.905 and 0.722, respectively).

#### Values and social beliefs

We also found positive and significant associations between CE and Self-Transcendent Beliefs (*r* = 0.45), Schwartz's Self-Transcendent Values (*r* = 0.34), Purpose in Life (*r* = 0.36) and Spirituality (*r* = 0.37).^3^ Fail-safe *N* tests returned values of 3,046, 206, 2,182, and 489 (respectively). In addition, all *p*-values of the rank correlations and Egger's regression tests were non-significant (*p* = 0.483 and 0.320; *p* = 0.083 and 0.133; *p* = 0.061 and 0.309; *p* = 0.083 and 0.212, respectively, for Self-Transcendent Beliefs, Values, Purpose in Life, and Spirituality), indicating and absence of publication bias for all analyses in this dimension.

#### Empowerment

For the final dimension, we found that CE was significantly associated with all the variables included. First, it was associated with Vitality (*r* = 0.24) with robust results and no evidence of publication bias (*N*fs = 147; rank correlation and regression test *p* = 0.483, and 0.122, respectively). The same was the case for its association with wellbeing (*r* = 0.32; *N*fs = 4,588; rank correlation and regression test *p* = 0.903, and 0.109, respectively), Self-Esteem (*r* = 0.15), Collective Efficacy (*r* = 0.46), and Collective Self-Esteem (*r* = 0.42), revealing stronger associations for variables at the collective level (i.e., Collective Self-Esteem) than the individual level (i.e., Self-Esteem). However, analyses revealed some indication of possible asymmetry in the funnel plot for the variables Collective Efficacy and Collective Self-Esteem, since the Egger's regression test was significant in both cases (*p*s <0.001) but not the rank correlation tests (*p* = 0.381 and 0.239, respectively). In the case of Self-Esteem, finally, both tests were significant (*p* = 0.017 and *p* < 0.001), suggesting strong asymmetry in the funnel plot.

### Total effect sizes, real correlations and future predictions

A further analyses of the total meta-analyzed effect sizes revealed that, from the 21 outcome variables analyzed in this study, 54.54% of them showed large effects (involving *k*_effectsizes_ = 146; *N* = 282710), around 30% were medium (involving *k*_effectsizes_ = 26; *N* = 8758), and around 13% were small (involving *k*_effectsizes_ = 5; *N* = 829). In addition, and as it was expected, the largest effects were found in the associations with proximal outcomes—also considered validity criteria—with variables such as Self-Transcendent Emotions and Fusion of Identity. Conversely, the weakest associations, were with Self-Esteem (individual), and only two associations were non-significant: those with Negative Emotions and with Negative Emotional Climate. In addition, an examination of the pooled effects from the real correlations (i.e., rhos; Table 3) suggests that the underlying relationship between CE and outcome variables is indeed stronger in all cases but, once again, non-significant with Negative Emotions and Negative Emotional Climate.

Finally, we conducted 80% CI prediction intervals (Riley et al., 2011), which correspond to an estimation where the true outcomes would fall in hypothetical new study from the population of studies. The results (Table 3) indicate that, with the exception of the relationship between CE and Negative Emotions, Negative Emotional Climate and Self-Esteem, all relationships in future studies would be positive and significant. In other words, that future studies should indeed reveal significant associations and among those, the majority should be of medium or high effect size.

### Moderation analyses

This section presents the results of the analyses of potential moderators in each dependent variable dimension. All tables reporting moderations analysis were included in Supplementary material.

#### Type of gathering

This analysis allows us to examine the specific association of CE with the outcomes during different type of collective gatherings (see Table 4). Regarding the type of event attended by participants (1 = Demonstration; 2 = Celebration; 3 = Religious event), results revealed similar effects for all dependent variables (*r*_avg_ = 0.40, 0.38, and 0.38, respectively) and that residual heterogeneity was decreased noticeably in 11 out of the 12 associations explored. In addition, and while there were no significant differences in the levels of the mediator for the analyses, we found a positive and significant association of CE and Negative Emotions [*r* = 0.14, 95% CI (0.01, 0.27); *k* = 8; *N* = 755], which was previously non-significant in the main results. Finally, it is worth noting that the relationship between CE and outcome variables did not change dramatically across the types of rituals; in fact, there were only significant differences in the association with Collective Efficacy. Specifically, CE associates with this variable more strongly in across Celebrations (*r* = 0.52) than Demonstrations (*r* = 0.38).

**Table 4:** Pooled correlations between collective effervescence and criterion variables moderated by type of collective gathering.

		**Residual heterogeneity**	**Test of moderators**			**Effect size**
**Dimension**	**Variable**	***QE*(*df*)**	***QM*(*df*)**	***k***	***N***	***r* (95% CI)**
**Proximal outcomes**						
Individual emotions	Arousal	*QE*(10) = 148.122, *p* < 0.001	*QM*(1) = 0.146, *p* = 0.703	8	622	0.413 (0.222, 0.603)
				4	1,013	0.474 (0.221, 0.727)
				2	46,681	
	Negative emotions	*QE*(11) = 47.373, *p* < 0.001	*QM*(1) = 2.915, *p* = 0.088	8	755	0.139 (0.004, 0.273)
				5	1,163	−0.041 (−0.191, 0.01)
				1	110	
	Positive emotions	*QE*(19) = 213.662, *p* < 0.001	*QM*(1) = 0.792, *p* = 0.373	11	3,754	0.585 (0.466, 0.705)
				10	1,970	0.508 (0.386, 0.629)
				1	110	
	ST emotions	*QE*(14) = 149.430, *p* < 0.001	*QM*(1) = 0.010, *p* = 0.922	9	3,516	0.579 (0.458, 0.701)
				7	1,714	0.571 (0.444, 0.697)
				1	110	
Communal sharing	Ingroup ID	*QE*(11) = 74.172, *p* < 0.001	*QM*(1) = 0.205, *p* = 0.651		576	0.517 (0.380, 0.655)
				6	1,501	0.472 (0.33, 0.611)
				1	1,176	
	FI pictorial	*QE*(9) = 46.328, *p* < 0.001	*QM*(1) = 2.306, *p* = 0.129	6	249	0.434 (0.286, 0.581)
				5	1,255	0.283 (0.157, 0.410)
				–	–	
	Social support	*QE*(9) = 109.526, *p* < 0.001	*QM*(1) = 0.906, *p* = 0.341	1	213	
				8	2230	0.363 (0.248, 0.479)
				3	1692	0.257 (0.071, 0.443)
**Distal outcomes**						
Social integration	Ingroup commitment	*QE*(4) = 1471.167, *p* < 0.001	*QM*(1) = 0.075, *p* = 0.784	3	911	0.340 (0.182, 0.499)
				1	409	
				3	122642	0.371 (0.221, 0.521)
Social values and beliefs	Purpose in life	*QE*(6) = 185.414, *p* < 0.001	*QM*(1) = 0.068, *p* = 0.794	3	2918	0.329 (0.083, 0.575)
				6	1602	0.369 (0.197, 0.541)
				-	-	
Empowerment	Wellbeing	*QE*(13) = 142.684, *p* < 0.001	*QM*(1) = 0.232, *p* = 0.630	5	3421	0.346 (0.168, 0.524)
				10	2611	0.294 (0.183, 0.406)
				2	156	
	Collective efficacy	*QE*(5) = 8.569, *p* = 0.128	*QM*(1) = 5.169, *p* = 0.023	3	342	0.367 (0.256, 0.478)
				4	1129	0.519 (0.449, 0.588)
				–	–	
	Collective self-esteem	*QE*(4) = 31.835, *p* < 0.001	*QM*(1) = 0.466, *p* = 0.495	3	288	0.383 (0.128, 0.639)
				3	1099	0.501 (0.280, 0.723)
				1	110	

*k* is the number of studies and *N* the number of participants included in the analysis. *r* (95% CI) indicates pooled Pearson's *r*s and their 95% confidence intervals. *QE*(*df* ) indicate the *Q* test of the residual heterogeneity test (i.e., after the moderation) and its degrees of freedom; *QM*(*df* ) indicate the *Q* test of comparison between the effect sizes between the levels of the moderator and its degrees of freedom. Moderator levels of type of gathering are 1 = Demonstration, 2 = Celebration, and 3 = Religious event. Dashes indicate that no study included the level and black spaces indicate that a given level was excluded due to no reaching the minimum condition for moderation analyses (i.e., *k* = 3).

Further, when the gathering in question was a demonstration (e.g., such as a political demonstration due in favor of changes in the political system, or due to the international women's day) (see Table 5), we found positive and significant associations of CE with a series of variables that are shown to produce and/or sustain collective action in different forms. In detail, CE was positively and significantly associated to Collective Identity (*r* = 0.52), Collective Efficacy (*r* = 0.37), Social Beliefs (*r* = 0.43) and specifically, on Negative Emotions, as previously mentioned. Overall, this supports the hypothesis that demonstrations are socialization instances that fuel factors conducive to long-term participation in collective action.

**Table 5:** Pooled correlations between collective effervescence on factors conductive to collective action in demonstrations.

			**Effect sizes**	
**Factors conductive to collective action**	***k***	***N***	***r* (95% CI)**	**Interpretation**
Group identification	7	576	0.517 (0.376, 0.657)	Large
Collective efficacy	3	342	0.371 (0.209, 0.533)	Large
Negative emotions (e.g., anger)	8	755	0.139 (0.004, 0.273)	Small
Self-transcendent emotions (e.g., awe)	9	3,516	0.582 (0.468, 0.695)	Large
Morality (self-transcendent beliefs)^a^	2	3,338	0.434 (0.046, 0.823)	Large

*k* is the number of studies and *N* the number of participants included in the analysis. *r* and (95% CI) indicate pooled Pearson's *r*s and their 95% confidence intervals. Factors conductive to collective action are integrated from the present article as well as different reviews (e.g., Agostini and van Zomeren, 2021; Akfirat et al., 2021). ^a^ While the analysis shows a large pooled effects size (as well in the direct affects), this relationship should be taken with cation due to the small amount of studies (i.e., *k* = 2).

#### Study design

When the effect sizes of the association between CE and outcome variables were moderated by the type of design (i.e., 1 = Cross-sectional; 2 = Longitudinal; Supplementary Table S1), results showed there was only a strong decrease of heterogeneity in the association with Negative Emotions and Ingroup Identity (extended); for the rest of the association, heterogeneity levels remained similar. Regarding the test between the levels of this moderator, on the other side, analyses revealed significant differences on Negative and Positive Emotions and Ingroup Identity (extended), indicating stronger associations of the variables with CE in cross-sectional studies in the case of Negative and Ingroup Identity, while the opposite was true for Positive Emotions (i.e., in longitudinal studies). Considering longitudinal studies as more supportive of the idea that CE is a cause of the outcomes, the fact that these kind of studies have similar or stronger effects (compared to cross-sectional ones) reaffirms the relevance of CE as an explanatory process.

#### CE measurement scales

Using as a moderation the different scales of CE (1 = PES; 2 = Positive Intense Emotionality: 3 = other scales such as State of Collective Effervescence and Emotional Entrainment; Supplementary Table S2), we found noticeable decreases of the levels of heterogeneity in 5 of the 6 associations that were performed. Comparing the levels of the mediator, we only found a difference between the PES and Positive Emotionality measure of CE for the association with Ingroup ID, with a lower association for the latter.

#### PES scale forms

In studies that included the PES scale as a measure of CE (*k* = 28; Supplementary Table S3), we found overall similar effects for the short (1) and long (2) forms of scale and decreases in the levels of heterogeneity in only 3 of the 7 associations we explored. In addition, we found no differences in the levels of the mediator in the association of CE with dependent variables. As a whole, these results support the validity of the short version of the PES scale.

#### Age, gender and cultural regions

Finally, meta-regression analyses (Supplementary Tables S4, S5), showed that both age and gender had an influence in the relationship between CE and dependent variables. Specifically, age positively affected the relationship with Spirituality and Wellbeing, while gender affected the relationship with Positive Emotions and Climate, the verbal measure of Fusion of Identity (positively), and Social Support (negatively). The analysis considering the cultural values, analyses revealed that national-level scores of Individualism-Collectivism influenced the association with Positive Emotions and Negative Climate (negatively), while Power Distance Index did it so with Positive Climate (positively) and Collective Efficacy (negatively).

## Discussion

Overall, CE was significantly but heterogeneously associated with, and predicted, the vast majority of criterion variables. More specifically, observed effects were stronger for proximal variables, compared to the distal ones. Confidence intervals excluded zero and Fail-safe *N*s were usually ten times larger than the number of included studies (between 99 and thousands of non-significant studies were necessary to nullify effect sizes). In addition, rank correlations and Egger's regressions suggested a lack of asymmetry in the funnel plots in 20 out of the 21 analyses performed and we found similar overall effects concerning the methodology (i.e., design and scale used to measure CE). Altogether, these findings suggest little evidence of publication bias and overall robust results.

First, CE was found to be related to proximal or immediate emotional outcomes: General Arousal, Positive Emotions, and Self-Transcendent Emotions. A strong result was found for General Arousal, which was associated with CE with a large effect size. These results confirmed that this variable was associated with a proximal or immediate intensification of emotions. CE was also strongly associated with positive emotions, supporting the “joy of gathering” or the essentially positive affective nature of effervescent states (Moscovici, 1988/1993; see also Ehrenreich, 2007). In addition, and consistent with Fiske (1992) and Haidt's (2012) proposals, CE was strongly associated with emotions that transcend the individual self (i.e., self-transcendent emotions). No relationship was found between CE and Negative Emotions. This may be partly explained by the fact that events involving clear and intense negative affectivity (e.g., funerary rituals) were not investigated. However, moderation analyses revealed that this relationship was significant in studies involving sociopolitical demonstrations, in which anger frequently played an important role. Thus, rather than assuming a non-existent relationship, it is safe to conclude that CE is indeed associated with individual negative emotions when they are salient and strong, in line with Durkheim's ideas.

Second, CE was associated with immediate outcomes related to various manifestations of communal sharing. It was strongly, albeit heterogeneously, associated with Social Support and with Identification with the Ingroup. Its association with Identity Fusion was also strong, confirming that CE has the potential to blur boundaries between the individual and the collective self. Together with the previous results, these findings support the view that, through CE, collective gatherings enhance a social or collective identification, social integration among participants, and as a long-term outcome social belongingness cohesion (Durkheim, 1912/1915; Collins, 2004). In this sense, CE was also associated with long-term self-investment in the ingroup (i.e., psychological commitment, and identification with the extended ingroup).

Third, CE was not only associated with Self-Transcendent Emotions but also with Spirituality or Self-Transcendent Beliefs and Values, with large effect sizes. These results confirm that this predictor redirects attention and reflection outwards and beyond the individual self, and by this token, constitutes a factor that can lead to the experience of self-transcendence (Van Cappellen and Rimé, 2014; Yaden et al., 2017). Findings support the view that CE puts people participating in collective events in contact with values and ideals—with the sacred (Durkheim, 1912/1915; Moscovici, 1988/1993; Gabriel et al., 2020). Specifically, CE was associated with the agreement with other-oriented values or the wellbeing of significant others, as well as with universalism or ideals of wellbeing and justice for all (i.e., Self-Transcendent Values; Schwartz, 2012). Furthermore, CE was associated with attributions of a Purpose in Life and beliefs that the world is just and has meaning. These effects were, respectively, large and medium. They confirm that rituals and collective encounters can be a source of attributions of positive meaning to life.

Fourth, CE was found to have medium and large effect-size associations with Empowerment-related constructs. It was indeed positively associated with Self-Esteem, Self-Efficacy, and Psychological Wellbeing. These results are in line with the idea that this variable promotes wellbeing through positive individual and collective emotions, social integration, salience, and adhesion to values and beliefs (Páez et al., 2015; Wlodarczyk et al., 2020). It is important to note that CE is more strongly connected to variables that function at collective levels (i.e., Collective Efficacy and Collective Self-Esteem) and that its effects relate to the ingroup reality, rather being related to intergroup relations (see Niedenthal and Brauer, 2012). Finally, results revealed that participation in effervescent collective gatherings was associated with a medium-size effect with vital energy, a finding that is consistent with evidence indicating that social integration enhances physical wellbeing (Larson, 1990; Holt-Lunstad et al., 2015; Liu et al., 2018). In short, people reinvigorate and replenish themselves through their experience of CE (Durkheim, 1912/1915; Moscovici, 1988/1993).

Fifth, CE was associated with Positive Collective Emotions with a medium effect size. This is an important finding since it supports the idea that CE helps to build an enduring shared mood (Collins, 2004) or long-term collective affect orientation (von Scheve et al., 2017) and that its effects are not limited to the creation of short-term emotional atmosphere. In addition, consistently with individual short-term emotional reactions, CE was not associated with Negative Emotional Climate.

Regarding moderation analyses, it is important to indicate that they could explain only a small part of the high level of heterogeneity observed in the effects.^4^ The analyses comparing design types found that cross-sectional studies had significant effect sizes which were similar, although slightly larger than those found in longitudinal studies—from the three differences found, in two cross-sectional showed larger effects. Longitudinal studies supported the view that this variable is an antecedent and potential cause of short-term increased emotionality (i.e., Total Emotionality), Positive Emotions, and Self-Transcendent Emotions, as well as enhanced Social Integration. In addition, it supports the idea that CE builds Positive Emotional Climate and long-term social cohesion, that it is related to a higher agreement with Self-Transcendent Beliefs and Values, and reinforcing individual and collective Empowerment, including Vitality.

The results of the moderation analyses focusing on the type of collective gathering are consistent with Durkheim's ideas that the effects are similar in collective gatherings with different content and valence, as well as in religious and secular rituals. They also revealed that Negative Emotions were associated with CE in the case of collective gatherings with mixed content and valence, such as sociopolitical demonstrations. Furthermore, the associations between CE and positive personal emotions were strong in events with positive content and valence, such as celebrations, thereby revealing convergent patterns.

The moderation analysis based on the type of scale revealed that associations were large for every scale assessing CE, although slightly stronger when it was measured with the PES scale, as well as with scales that only emphasized mutual emotional stimulation or connecting with others. However, as indicated previously, both the heterogeneity of measurements and the lack of a larger number of studies make it difficult to draw clear and solid conclusions. In contrast, subsequent analyses comparing the short and long forms of the PES scale supported the usefulness of the PES short version scale (Wlodarczyk et al., 2020), since the two forms yielded similar effect sizes.

Concerning the relevance of participation in collective behaviors on socially and politically relevant phenomena, CE during demonstrations correlates to several outcomes similarly as it is found in other forms of collective gatherings. What is more, CE during demonstrations was associated with factors favorable to social movements like social or Collective Identity. Together with Agostini and van Zomeren's (2021) meta-analysis, this identification predicted participation in collective action, and thus, it is possible to assume CE as a catalyzer for collective action. Taking into account Akfirat et al.'s (2021) meta-analysis on identity and network-based social movements, we also see a strong relationship between social identification and participation in collective action. The relationship between participation in collective action and identification with emergent groups was also found to be stronger than identification with pre-existing groups. Thus, identification with an emerging group (e.g., protest groups, opinion groups), better predicts participation in collective action than identification with pre-existing social groups (e.g., nations, religious groups, ideological groups, etc.). Participation in demonstrations appears as a key factor to promote this emergent politicized collective identity. Furthermore, CE also correlates with Collective Efficacy, which is a strong predictor of participation in collective action and associates strongly with participation in collective behavior (see also van Zomeren et al., 2008).

In the same vein, CE during demonstrations strongly correlates with Positive Emotions, and Self-Transcendent Emotions (e.g., hope) that play a role in social mobilization (Páez et al., 2013). In addition, CE correlates with Negative Emotions (e.g., anger), and these types of emotions are conducive to participation in collective behavior and social movements. According to van Zomeren et al.' (2008) meta-analysis, the emotional or affective experience of injustice—which is also strongly associated with collective behaviors—has stronger effects on collective action than non-affective perceived injustice, which also goes in line with Smith et al.'s (2012) review underlying the emotional (i.e., compared the cognitive) facet of deprivation. Finally, and as it has been shown elsewhere (e.g., Agostini and van Zomeren, 2021), the age, gender, as well as cultural regions can have an effect in the association with several outcomes included in this meta-analysis.

To conclude, this article shows that CE is not only associated, but also longitudinally predicts, positive outcomes, particularly when the measure is not limited to feeling intense positive emotions, but rather emphasizes interaction and emotional connection with others. The results thus suggest that it can affect the participants of a collective event in a significant form: their emotions, their power to act, their social positioning, as well as their beliefs and values. It is safe to conclude, therefore, that CE was not a myth that came out of Durkheim's imagination.

### Future perspectives

In support of what was advanced by Moscovici (1988/1993) and Collins (2004), CE emerges from the reviewed results as a powerful tool for transforming individuals. It represents an instrument that brings individuals together, gives them self-confidence, and infuses them with values and beliefs. However, and considering the “positivity” of the results presented here, we must not overlook the fact that the same effects can also lead to negative consequences. To illustrate, during the decades before the Second World War, totalitarian parties perfectly understood the use they could make of such a tool (i.e., exploitations of collective gatherings). As the abundant film archives attest, they did indeed make an immoderate use of it. It is not utopian to think that, in the absence of this tool, these parties would not have had the impact they had for humanity's misfortune. At the very same time, scientists had left the questions of collective gatherings and collective emotions in the shade. Now, that CE and its effects are moving beyond the realm of mere theorization and are taking up a place among empirical findings, there are compelling reasons for scientific work to catch up and develop knowledge on the scope and limits of the collective tool. In particular, future research will have the task of specifying to what extent and under what conditions CE constitutes an instrument of persuasion. We currently do not know what the degree of plasticity of individuals in a collective situation is, and many questions arise in this regard. For instance, to what extent are the participants in a collective event likely to incorporate ideas, beliefs, or values that differ from those they previously held? What is the duration of the impact or effects of participating in a group situation? Are these effects of equal importance and duration with respect to emotions, motivation, social connections, and beliefs and values? Are there individual differences that make individuals more or less susceptible to the effects of collective situations? What resilience or resistance tools are available to participants in collective situations? These are just a few examples of the many questions that need to be investigated.

A burgeoning line of research has employed indicators of CE together with measures tapping into the effects of participation in collective gatherings. We were able to locate 59 studies of this type and subjected them to a meta-analysis. It assessed the extent to which indicators of CE were associated with the different types of effects mentioned in the conclusion of our literature review. The studies included in this meta-analysis covered a wide variety of populations and a wide range of collective events. They also involved diverse measures of CE and a broad array of instruments for the evaluation of the different classes of potential effects. Our results confirmed that most of the variables identified in our literature review were significantly associated with the measures of CE, often with large effect sizes. This review provides support to Durkheim's original theorizations and the available data here can shed light on the effects of collective gatherings of all types.

## Author contributions

DP and BR conceptualized and organized the present study and elaborated the first draft. JP and LZ conducted all the transformations and codification of studies, and the subsequent statistical analyses (with the supervision of DP), which in turn were reviewed by all authors. All authors reviewed and commented on the final version of this study. All authors contributed to the article and approved the submitted version.

## Funding

This research was supported by grants given to the research team Culture, Cognition and Emotion (Psicología Social CCE), the Spanish Ministry of Economy and Competitiveness ([Ref.: PSI2017-84145-P and Ref.: PID2020-115738GB-I00), the Basque Government (Ref.: GIC12/91 IT-666-13, IT1187-19, and IT1598-22]), and a post-doc grant from the UPV/EHU to JP (DOCBERRI 20/23).

## Conflict of interest

The authors declare that the research was conducted in the absence of any commercial or financial relationships that could be construed as a potential conflict of interest.

## Publisher's note

All claims expressed in this article are solely those of the authors and do not necessarily represent those of their affiliated organizations, or those of the publisher, the editors and the reviewers. Any product that may be evaluated in this article, or claim that may be made by its manufacturer, is not guaranteed or endorsed by the publisher.

## Footnotes

^1^It is important to point that Collins' approach is convergent with the fact that Durkheim agrees with Le Bon (2002) and Freud (1922) that, in a crowd or mass or collective gathering, the person acquires new qualities. These authors share that collective encounters, and crowds, transform people to think and act in ways they otherwise would not. During these states of collective encounters, beliefs, behaviors, and emotions become polarized, much more extreme, and much more similar (Moscovici, 1988/1993). Le Bon asserts that crowds, although they do not profess conventional morality, are capable of moral acts -sacrificial and selfless- far superior to those of the individual person. Freud asserts a similar idea. Durkheim will insist that collective encounters will affectively recharge moral ideals and norms. That is, all these authors agree that people in collective rituals and manifestations may act in more extreme and idealistic or moral ways-showing extreme altruism and sacrifice, but also extreme violence (Moscovici, 1988/1993).

^2^The *r* represents pooled Pearson's correlation from random-model meta-analysis.

^3^In the case of Just World (e.g., World Assumption Scale, as in Páez et al., 2015), the association was positive and significant (*r*_*pooled*_ = 0.27; *Q*_(1)_ = 0.083, *p* = 0.773) but the analysis was removed due to the amount of studies (*k* = 2).

^4^While undesirable, this is not unusual in meta-analyses; see Siegel et al. (2018).
