# Supplementary material for: Emotional processes, collective behavior, and social movements: A meta-analytic review of collective effervescence outcomes during collective gatherings and demonstrations
Source: Front Psychol. 2022 Aug 31;13:974683. doi: 10.3389/fpsyg.2022.974683 (PMC9473704; doi:10.3389/fpsyg.2022.974683)
Supplement: Supplementary file 1 [file Data_Sheet_1.pdf]

Supplementary Table S1.

Pooled Correlations between Collective Effervescence and Criterion Variables Moderated by Study Design.

Dimension	Variable	Residual Heterogeneity $QE(df)$	Test of Moderators $QM(df)$	*k*	*N*	Effect Size *r* [95% CI]
Proximal Outcomes						
Individual Emotions	Arousal	*QE*(12)=178.723, *p*<.001	*QM*(1)=3.052, *p*=.081	5	47311	.312 [.126, .499]
				9	1005	.525 [.377, .673]
	Negative Emotions	*QE*(12)=32.890, *p*=.001	*QM*(1)=5.619, *p*=.018	5	1023	.160 [.035, .284]
				9	1005	-.046 [-.162, .070]
	Positive Emotions	*QE*(20)=239.186, *p*<.001	*QM*(1)=4.196, *p*=.041	11	4320	.471 [.367, .576]
				11	1514	.628 [.521, .735]
	ST Emotions	*QE*(15)=171.261, *p*<.001	*QM*(1)=1.586, *p*=.208	9	4299	.535 [.434, .635]
				8	1041	.634 [.517, .750]
Communal Sharing	Ingroup ID	*QE*(12)=295.187, *p*<.001	*QM*(1)=0.231, *p*=.631	8	2349	.433 [.287, .578]
				6	904	.489 [.312, .666]
	FI Pictorial	*QE*(9)=26.905, *p*=.001	*QM*(1)=1.145, *p*=.285	3	609	.274 [.109, .440]
				8	895	.386 [.265, .507]
Distal Outcomes						
Social Integration	Ingroup ID (extended)	*QE*(6)=94.263, *p*<.001	*QM*(2)=22.539, *p*<.001	5	74221	.332 [.164, .500]
				2	918	.300 [.085, .516]
Social Values and Beliefs	Purpose in Life	*QE*(6)=274.021, *p*<.001	*QM*(1)=0.150, *p*=.698	5	3853	.375 [.205, .545]
				3	625	.316 [.070, .562]
	Wellbeing	*QE*(15)=158.084, *p*<.001	*QM*(1)=0.785, *p*=.376	8	4607	.280 [.168, .393]
				9	1581	.354 [.235, .473]
Empowerment	Collective Self-Esteem	*QE*(5)=35.954, *p*<.001	*QM*(1)=3.316, *p*=.069	3	762	.544 [.360, .729]
				4	735	.300 [.113, .487]

Note. *k* is the number of studies and *N* the number of participants included in the analysis. *r* [95% CI] indicates pooled Pearson's *r*s and their 95% confidence intervals. *QE*(*df*) indicate the *Q* test of the residual heterogeneity test (i.e., after the moderation) and its degrees of freedom; *QM*(*df*) indicate the *Q* test of comparison between the effect sizes between the levels of the moderator and its degrees of freedom. Moderator levels of type of gathering are 1 = Cross-sectional and 2 = Longitudinal design. The variables not included here were due to no having reached the minimum condition for moderation analyses (i.e., *k* = 3).

Supplementary Table S2.

Pooled Correlations between Collective Effervescence and Criterion Variables Moderated by CE Scale.

		Residual Heterogeneity	Test of Moderators			Effect Size
Dimension	Variable	*QE*(*df*)	*QM*(*df*)	*k*	*N*	*r* [95% CI]
Proximal Outcomes						
	Ingroup ID	*QE*(12)=179.084, *p*<.001	*QM*(1)=5.132, *p*=.023	10	1522	.529 [.416, .643]
				4	1731	.286 [.110, .463]
Communal Sharing				-	-	
	Social Support	*QE*(8)=61.558, *p*<.001	*QM*(1)=0.121, *p*=.728	5	1232	.355 [.228, .482]
				2	1592	
				5	1311	.387 [.262, .512]
Distal Outcomes						
	Ingroup Commitment	*QE*(4)=1471.167, *p*<.001	*QM*(1)=0.075, *p*=.784	3	911	.340 [.182, .499]
				1	409	
				3	122642	.371 [.221, .521]
Social Integration	Ingroup ID (extended)	*QE*(5)=93.612, *p*<.001	*QM*(1)=0.158, *p*=.691	4	922	.314 [.118, .510]
				1	416	
				3	73801	.374 [.155, .593]
	Purpose in Life	*QE*(6)=107.546, *p*<.001	*QM*(1)=1.158, *p*=.282	5	3561	.296 [.124, .468]
Social Values and Beliefs				3	917	.442 [.240, .644]
	Wellbeing	*QE*(14)=120.920, *p*<.001	*QM*(1)=0.128, *p*=.721	12	5025	.328 [.224, .432]
Empowerment				1	495	
				4	668	.291 [.114, .467]

Note *k* is the number of studies and *N* the number of participants included in the analysis. *r* [95% CI] indicates pooled Pearson's *r*s and their 95% confidence intervals. *QE*(*df*) indicate the *Q* test of the residual heterogeneity test (i.e., after the moderation) and its degrees of freedom; *QM*(*df*) indicate the *Q* test of comparison between the effect sizes between the levels of the moderator and its degrees of freedom. Moderator

levels of type of gathering are 1 = PES and 2 = Positive Emotionality, and 3 = Other scale. The variables not included here were due to no having reached the minimum condition for moderation analyses (i.e., $k = 3$).

Supplementary Table S3.

Pooled Correlations between Collective Effervescence and Criterion Variables Moderated by PES Scale Length.

		Residual Heterogeneity	Test of Moderators			Effect Size
Dimension	Variable	*QE*(*df*)	*QM*(*df*)	*k*	*N*	*r* [95% CI]
Proximal Outcomes						
	Arousal	*QE*(11)=161.327, *p*<.001	*QM*(1)=0.848, *p*=.357	8	836	.384 [.212, .556]
				5	909	.516 [.295, .736]
	Negative Emotions	*QE*(11)=70.612, *p*<.001	*QM*(1)=0.547, *p*=.460	7	771	.012 [-.143, .166]
Individual Emotions				6	1107	.099 [-.074, .272]
	Positive Emotions	*QE*(18)=165.504, *p*<.001	*QM*(1)=2.827, *p*=.093	13	4386	.616 [.522, .710]
				7	1200	.474 [.337, .610]
	ST Emotions	*QE*(15)=156.210, *p*<.001	*QM*(1)=0.462, *p*=.496	12	4431	.592 [.498, .686]
				5	909	.528 [.367, .688]
	Ingroup ID	*QE*(8)=45.364, *p*<.001	*QM*(1)=0.682, *p*=.409	4	462	.487 [.342, .633]
Communal Sharing				6	1060	.568 [.444, .691]
	FI Pictorial	*QE*(9)=27.751, *p*=.001	*QM*(1)=0.668, *p*=.414	7	871	.319 [.199, .439]
				4	633	.410 [.229, .590]
Distal Outcomes						
Empowerment	Wellbeing	*QE*(10)=104.703, *p*<.001	*QM*(1)=0.049, *p*=.826	7	4116	.334 [.188, .481]
				5	909	.307 [.112, .502]

Note. *k* is the number of studies and *N* the number of participants included in the analysis. *r* [95% CI] indicates pooled Pearson's *r*s and their 95% confidence intervals. *QE*(*df*) indicate the *Q* test of the residual heterogeneity test (i.e., after the moderation) and its degrees of freedom; *QM*(*df*) indicate the *Q* test of comparison between the effect sizes between the levels of the moderator and its degrees of freedom. Moderator levels of type of gathering are 1 = Short and 2 = Long format of the PES Scale. The variables not included here were due to no having reached the minimum condition for moderation analyses (i.e., *k* = 3).

Supplementary Table S4.

Pooled Correlations between Collective Effervescence and Criterion Variables Moderated by Age and Women Proportion.

		Residual Heterogeneity	Test of Moderators				Effect Size
Dimension	Variable	*QE*(*df*)	*QM*(*df*)	*k*	*N*	Estimate	*r* [95% CI]
Proximal Outcomes							
	Arousal	*QE*(10)=128.862, *p*<.001	*QM*(2)=2.749, *p*=.253	13	1745	Int.	-0.636 [-2.163, 0.891]
						β_{Age}	0.006 [-0.011, 0.023]
						β_{Women}	0.013 [-0.003, 0.029]
	Negative Emotions	*QE*(11)=64.891, *p*<.001	*QM*(2)=0.103, *p*=.950	14	2028	Int.	0.200 [-0.749, 1.150]
						β_{Age}	-0.002 [-0.015, 0.011]
Individual Emotions						β_{Women}	-0.002 [-0.012, 0.009]
	Positive Emotions	*QE*(19)=129.217, *p*<.001	*QM*(2)=13.866, *p*<.001	22	5834	Int.	-0.196 [-0.624, 0.231]
						β_{Age}	0.007 [-0.000, 0.014]
						β_{Women}	0.008 [0.004, 0.013]
	ST Emotions	*QE*(14)=97.876, *p*<.001	*QM*(2)=3.909, *p*=.142	17	5340	Int.	-0.144 [-0.908, 0.620]
						β_{Age}	0.009 [-0.000, 0.019]
						β_{Women}	0.006 [-0.002, 0.014]
	Ingroup ID	*QE*(10)=63.078, *p*<.001	*QM*(2)=0.408, *p*=.815	13	2077	Int.	0.340 [-0.191, 0.871]
						β_{Age}	0.001 [-0.009, 0.011]
						β_{Women}	0.002 [-0.005, 0.009]
	FI Verbal	*QE*(2)=0.631, *p*=.730	*QM*(2)=6.328, *p*=.042	5	1031	Int.	0.440 [0.197, 0.684]
						β_{Age}	0.002 [-0.002, 0.005]
Communal Sharing						β_{Women}	0.004 [0.001, 0.006]
	FI Pictorial	*QE*(8)=20.816, *p*=.008	*QM*(2)=3.050, *p*=.218	11	1504	Int.	-0.641 [-2.234, 0.951]
						β_{Age}	-0.005 [-0.004, 0.037]
						β_{Women}	-0.006 [-0.008, 0.023]
	Social Support	*QE*(7)=47.714, *p*<.001	*QM*(2)=4.170, *p*=.124	10	2859	Int.	0.578 [0.261, 0.894]
						β_{Age}	0.002 [-0.003, 0.007]
						β_{Women}	-0.006 [-0.012, -0.000]
Distal Outcomes							
	Negative Climate	*QE*(2)=8.746, *p*=.013	*QM*(2)=2.895, *p*=.235	5	1357	Int.	0.536 [-0.174, 1.245]

						β_{Age}	-0.015 [-0.034, 0.003]
						β_{Women}	-0.002 [-0.011, 0.007]
Collective Emotions	Positive Climate			4	1159	Int.	0.386 [-0.007, 0.779]
		QE(1)=1.351, *p*=.245	*QM*(2)=18.990, *p*<.001			β_{Age}	-0.030 [-0.044, -0.015]
						β_{Women}	0.009 [0.004, 0.015]
	Ingroup Commitment	*QE*(3)=23.403, *p*<.001	*QM*(2)=3.415, *p*=.181	6	50766	Int.	0.757 [0.310, 1.205]
						β_{Age}	-0.002 [-0.010, 0.007]
Social Integration						β_{Women}	-0.006 [-0.012, 0.001]
	Ingroup ID (extended)	*QE*(3)=20.831, *p*<.001	*QM*(2)=2.546, *p*=.280	6	1436	Int.	0.964 [0.174, 1.755]
						β_{Age}	-0.004 [-0.012, 0.005]
						β_{Women}	-0.009 [-0.022, 0.004]
	ST Beliefs	*QE*(2)=46.748, *p*<.001	*QM*(2)=2.373, *p*=.305	5	4231	Int.	-1.302 [-3.557, 0.954]
						β_{Age}	0.023 [-0.010, 0.056]
						β_{Women}	0.015 [-0.004, 0.035]
	ST Values	*QE*(1)=0.059, *p*=.808	*QM*(2)=2.640, *p*=.267	4	1103	Int.	2.988 [-0.495, 6.471]
						β_{Age}	-0.048 [-0.106, 0.011]
Social Values and Beliefs						β_{Women}	-0.022 [-0.054, 0.09]
	Purpose in Life	*QE*(5)=181.769, *p*<.001	*QM*(2)=0.016, *p*=.992	8	4478	Int.	0.350 [-0.271, 0.971]
						β_{Age}	0.001 [-0.014, 0.015]
						β_{Women}	-0.000 [-0.007, 0.006]
	Spirituality	*QE*(2)=4.017, *p*=.134	*QM*(2)=7.503, *p*=.023	5	1416	Int.	-5000 [-9.378, -0.621]
						β_{Age}	0.015 [0.001, 0.030]
						β_{Women}	0.075 [0.015, 0.135]
	Vitality	*QE*(2)=3.950, *p*=.139	*QM*(2)=1.189, *p*=.552	5	1411	Int.	-0.619 [-3.414, 2.176]
						β_{Age}	0.013 [-0.022, 0.048]
						β_{Women}	0.007 [-0.021, 0.036]
Empowerment	Wellbeing	*QE*(14)=82.063, *p*<.001	*QM*(2)=5.120, *p*=.077	17	6188	Int.	-0.123 [-0.556, 0.310]
						β_{Age}	0.008 [0.001, 0.016]
						β_{Women}	0.003 [-0.002, 0.008]
	Collective Efficacy	*QE*(4)=11.865, *p*=.018	*QM*(2)=1.280, *p*=.527	7	1471	Int.	1.047 [0.026, 2.068]

					β_{Age}	-0.007 [-0.021, 0.007]
					β_{Women}	-0.005 [-0.015, 0.005]
Collective Self-Esteem	*QE*(4)=39.796, *p*<.001	*QM*(2)=0.828, *p*=.661	7	1497	Int.	0.781 [-0.051, 1.612]
					β_{Age}	-0.004 [-0.018, 0.011]
					β_{Women}	-0.004 [-0.014, 0.006]

Note. *k* is the number of studies and *N* the number of participants included in the analysis. *r* [95% CI] indicates pooled Pearson's *r*s and their 95% confidence intervals. *QE*(*df*) indicate the *Q* test of the residual heterogeneity test (i.e., after the moderation) and its degrees of freedom; *QM*(*df*) indicate the *Q* test of comparison of the effects of the mean age of the participants in each study (i.e., β_{Age}) and the percentage of women of each study (i.e., β_{Women}). Int., represents the slope of the analysis. The variable not included here was due to no having reached the minimum condition for moderation analyses (i.e., $k > 3$).

Supplementary Table S5.

Pooled Correlations between Collective Effervescence and Criterion Variables Moderated by National Levels Of Power Distance Index and Individualism.

		Residual Heterogeneity	Test of Moderators				Effect Size
Dimension	Variable	*QE*(*df*)	*QM*(*df*)	*k*	*N*	Estimate	*r* [95% CI]
Proximal Outcomes							
Individual Emotions	Arousal	*QE*(10)=153.976, *p*<.001	*QM*(2)=0.505, *p*=.777	13	1745	Int.	1.576 [-1.584, 4.735]
						β_{PDI}	-0.018 [-0.069, 0.033]
						β_{IND}	-0.002 [-0.013, 0.010]
	Negative Emotions	*QE*(10)=53.271, *p*<.001	*QM*(2)=1.230, *p*=.541	13	1878	Int.	-1.131[-3.250, 0.987]
						β_{PDI}	0.020 [-0.018, 0.058]
						β_{IND}	0.000 [-0.009, 0.010]
	Positive Emotions	*QE*(17)=141.460, *p*<.001	*QM*(2)=5.929, *p*=.052	20	5586	Int.	1.234 [0.412, 2.056]
						β_{PDI}	-0.006 [-0.018, 0.007]
						β_{IND}	-0.006 [-0.011, -0.001]
	ST Emotions	*QE*(14)=158.974, *p*<.001	*QM*(2)=1.362, *p*=.506	17	5340	Int.	0.813 [-0.002, 1.629]
						β_{PDI}	-0.004 [-0.014, 0.006]
						β_{IND}	-0.000 [-0.007, 0.007]
Communal Sharing	Ingroup ID	*QE*(8)=51.783, *p*<.001	*QM*(2)=0.853, *p*=.653	11	1931	Int.	0.761 [-0.115, 1.636]
						β_{PDI}	-0.004 [-0.017, 0.008]
						β_{IND}	0.001 [-0.006, 0.007]
	FI Verbal	*QE*(3)=3.719, *p*=.293	*QM*(1)=2.804, *p*=.094	5	1031	Int.	1.686 [0.525, 2.847]
						β_{PDI}	-0.017 [-0.037, 0.003]
						β_{IND}	-
	FI Pictorial	*QE*(8)=14.313, *p*=.074	*QM*(2)=6.380, *p*=.041	11	1504	Int.	1.021 [0.045, 1.997]
						β_{PDI}	-0.010 [-0.021, 0.000]
						β_{IND}	-0.000 [-0.009, 0.008]
	Social Support	*QE*(3)=15.838, *p*=.001	*QM*(2)=1.867, *p*=.393	6	1648	Int.	-0.370 [-1.418, 0.678]
						β_{PDI}	0.005 [-0.006, 0.017]
						β_{IND}	0.007 [-0.005, 0.018]

Distal Outcomes							
	Negative Climate	*QE*(2)=2.100, *p*=.350	*QM*(2)=17.867, *p*<.001	5	1357	Int.	0.653 [-0.239, 1.545]
						β_{PDI}	-0.004 [-0.020, 0.012]
Collective Emotions						β_{IND}	-0.007 [-0.011, -0.003]
	Positive Climate	*QE*(1)=2.301, *p*=.129	*QM*(2)=11.982, *p*=.003	4	1159	Int.	1.994 [3.443, -0.545]
						β_{PDI}	0.034 [0.009, 0.058]
						β_{IND}	0.004 [-0.001, 0.010]
	Ingroup Commitment	*QE*(2)=4.217, *p*=.121	*QM*(1)=3.044, *p*=.081	4	1320	Int.	0.715 [0.329, 1.101]
						β_{PDI}	-0.007 [-0.014, 0.001]
Social Integration						β_{IND}	-
	Ingroup ID (extended)	*QE*(4)=56.812, *p*<.001	*QM*(2)=0.267, *p*=.875	7	1943	Int.	0.635 [-0.804, 2.074]
						β_{PDI}	-0.003 [-0.016, 0.009]
						β_{IND}	-0.003 [-0.017, 0.012]
	ST Beliefs	*QE*(2)=32.266, *p*<.001	*QM*(1)=0.800, *p*=.371	4	3736	Int.	-0.034 [-1.190, 1.122]
						β_{PDI}	0.008 [-0.010, 0.026]
						β_{IND}	-
	ST Values	*QE*(2)=2.695, *p*=.260	*QM*(1)=0.004, *p*=.951	4	1103	Int.	0.348 [-0.078, 0.774]
						β_{PDI}	-0.000 [-0.006, 0.005]
Social Values and Beliefs						β_{IND}	-0.001 [-0.009, 0.007]
	Purpose in Life	*QE*(2)=0.179, *p*=.914	*QM*(2)=1.464, *p*=.481	5	3561	Int.	0.543 [-0.208, 1.294]
						β_{PDI}	-0.002 [-0.009, 0.005]
						β_{IND}	-0.003 [-0.010, 0.003]
	Spirituality	*QE*(2)=10.765, *p*=.005	*QM*(2)=0.534, *p*=.766	5	1416	Int.	1.285 [-1.216, 3.786]
						β_{PDI}	-0.009 [-0.032, 0.015]
						β_{IND}	-0.007 [-0.029, 0.016]
	Vitality	*QE*(3)=6.411, *p*=.093	*QM*(1)=0.335, *p*=.563	5	1411	Int.	0.372 [-0.099, 0.844]
						β_{PDI}	-0.002 [-0.009, 0.005]
Empowerment						β_{IND}	-
	Wellbeing	*QE*(9)=109.609, *p*<.001	*QM*(2)=0.009, *p*=.995	12	5025	Int.	0.369 [-1.317, 2.054]
						β_{PDI}	-0.001 [-0.018, 0.016]

					β_{IND}	-0.000 [-0.014, 0.014]
Collective Efficacy	*QE*(5)=9.856, *p*=.079	*QM*(1)=4.163, *p*=.041	7	1471	Int.	2.041 [0.533, 3.549]
					β_{PDI}	-0.027 [-0.053, -0.001]
					β_{IND}	-

Note. *k* is the number of studies and *N* the number of participants included in the analysis. *r* [95% CI] indicates pooled Pearson's *r*s and their 95% confidence intervals. *QE*(*df*) indicate the *Q* test of the residual heterogeneity test (i.e., after the moderation) and its degrees of freedom; *QM*(*df*) indicate the *Q* test of the effects of national-level cultural dimensions of Power Distance Index (i.e., β_{PDI}) and Individualism (i.e., β_{IND}). Int., represents the slope of the analysis. Dashes indicate that there were no values for country where the association was evaluated. The variable not included here was due to no having values in the country-level indicators.
